# Effects of pits of different ages on ethyl acetate and its metabolism-related microorganisms during strong-flavor Baijiu fermentation

**DOI:** 10.3389/fmicb.2025.1532869

**Published:** 2025-01-27

**Authors:** Jing Zhang, Yunxuan Duan, Yang Lin, Jing Chen, Jie Cheng, Chuan Song, Jincen Zuo, Suyi Zhang, Yong Zuo

**Affiliations:** ^1^College of Food and Biological Engineering, Chengdu University, Chengdu, China; ^2^Luzhou Laojiao Group Co., Ltd., Luzhou, China; ^3^College of Life Science, Sichuan Normal University, Chengdu, China; ^4^Luzhoulaojiao Postdoctoral Programme, Luzhou Laojiao Group Co., Ltd., Luzhou, China; ^5^Xichong County, Agricultural and Rural Bureau, Nanchong, China

**Keywords:** strong-flavor Baijiu, pits, ethyl acetate, microbial diversity, volatile flavor components

## Abstract

The esters are the most important flavor components in Baijiu as their species and content decide the style of Baijiu. During the formation of esters, pits play important roles. In this study, the main esters and their related microorganisms in different years of pits (5, 35 and 100 years) of strong-flavor Baijiu were comprehensively researched by headspace solid-phase microextraction gas chromatography mass spectrometry (HS-SPME-GC-MS) and amplicon sequencing. A total of 690 bacterial genera and 155 fungal genera were detected. The microbial composition of ZPs (fermented grains) from 100 years pit was the most abundant at the genus level. A total of 177 volatile flavor components were observed, including 80 esters, 42 alcohols, 21 acids, 10 ketones and 11 aldehydes. Ethyl acetate was the lowest and ethyl caproate was relatively high in 100 years pit. 15 genera, including *Lactobacillus*, *Pichia*, *Issatchenkia*, *Saccharomyces*, and *Aspergillus*, were positively related to the formation of four major esters and their precursors. The research demonstrated that 100 years pit was benefit for maintaining microbial diversity and controlling ethyl acetate. This study is helpful for understanding the microbial composition and succession in the fermentation process of strong-flavor Baijiu, and revealing the complex relationships between dominant genera, physicochemical properties and volatile flavor components.

## 1 Introduction

Baijiu, a unique Chinese alcoholic beverage with a long history and rich cultural significance, is one of the six distilled spirits in the world (Yang et al., 2023). Water and ethanol are the primary components of Baijiu, comprising approximately 98% of its content. The remaining 2% consists of trace flavor components, including alcohols, aldehydes, acids, and esters (Wang Z. et al., 2023). Among these, esters are the most important flavor components, contributing strong fruit, sweet, and milk flavors that define the style of Baijiu (Huang et al., 2022). To date, more than 500 esters have been detected in Baiju from various flavor types, with the four most important esters—ethyl caproate, ethyl acetate, ethyl lactate, and ethyl butyrate—accounting for approximately 90% of the total esters (Xu Y. Q. et al., 2022). Ethyl acetate, with a threshold of 32,600 μg/L, is one of the typical flavor components in Baijiu (Wei et al., 2023). Strong-flavor Baijiu, made from various grains, mainly sorghum as the raw material, and fermented with medium-high temperature Daqu as the saccharification starter, undergoes production processes including cooking, saccharification, fermentation, distillation, and storage. In this type of Baijiu, the content of ethyl acetate is typically lower than that of ethyl caproate, and long-term production practice has shown that the ratio of ethyl acetate to ethyl caproate in high-quality Baijiu ranges between 0.5 and 1. However, in recent years, the content of ethyl acetate has generally increased during the production of strong-flavor Baijiu in some enterprises, leading to issues such as incongruous aroma and an unremarkable style. Therefore, controlling the content of ethyl acetate has become an important issue in the production of strong-flavor Baijiu.

Ethyl acetate is produced through the fermentation of yeast, bacteria, and mold (Xu Y. Q. et al., 2022). At the beginning of the fermentation, with the assistance of acetyl-CoA synthetase (ACS), acetic acid is converted into acetyl-CoA in yeast cells. This is then further converted into ethyl acetate in the presence of alcohol acetyltransferases *Atf1p*, *Atf2p*, and *Lg Atf1p* (Kruis et al., 2018). During the middle and late stages of the fermentation, ethyl acetate is synthesized via an esterification reaction between acetic acid and ethanol, catalyzed by esterifying enzymes primarily secreted by yeast and mold (Cui et al., 2018). In addition, other microorganisms also produce small amounts of ethyl acetate during the fermentation (Li et al., 2017). However, as we know, Baijiu brewing involves complex techniques in an open environment, which leads to the introduction of a large number of microorganisms into the production of Baijiu through Daqu, pit mud, and environmental air (Mu et al., 2022). Thus, the growth and metabolism of the microorganisms can be affected by the environmental factors, brewing techniques, and microbial interactions.

In the past few years, various strategies have been proposed to explore the influence of Daqu, pits, and brewing techniques on microbial succession and main flavor components. Using headspace solid-phase microextraction with gas chromatography–mass spectrometry (HS-SPME–GC–MS), He et al. analyzed the flavor components of Daqu during storage. It was found that the flavor components gradually accumulated and that ethyl acetate presented the highest content after 3 months of Daqu storage (He et al., 2022). Xu et al. compared the fermented grains from new and old pits and found that the starch utilization rate was faster and the ethanol yield was higher in the old pit, while the acidity and ethyl acetate contents were higher in the new pit (Xu S. S. et al., 2022). Compared to traditional brewing techniques that mainly rely on individual operation, the levels of ethyl lactate, ethyl acetate, and ethyl caproate in Baijiu brewed using an automatic system are significantly reduced, mainly due to the fermentation activity of lactic acid bacteria, yeast, and mold (Wang X. S. et al., 2022). Whether adjusting the Daqu storage time, using pits of different ages, or changing brewing technology, the key to controlling ethyl acetate lies in regulating microorganisms. However, current research on ethyl acetate is primarily centered on Daqu, pits, and brewing technology, with limited focus and inconsistent direction. The metabolic pathways of ethyl acetate and the microorganisms involved in its metabolism need further analysis.

**Scheme 1:**
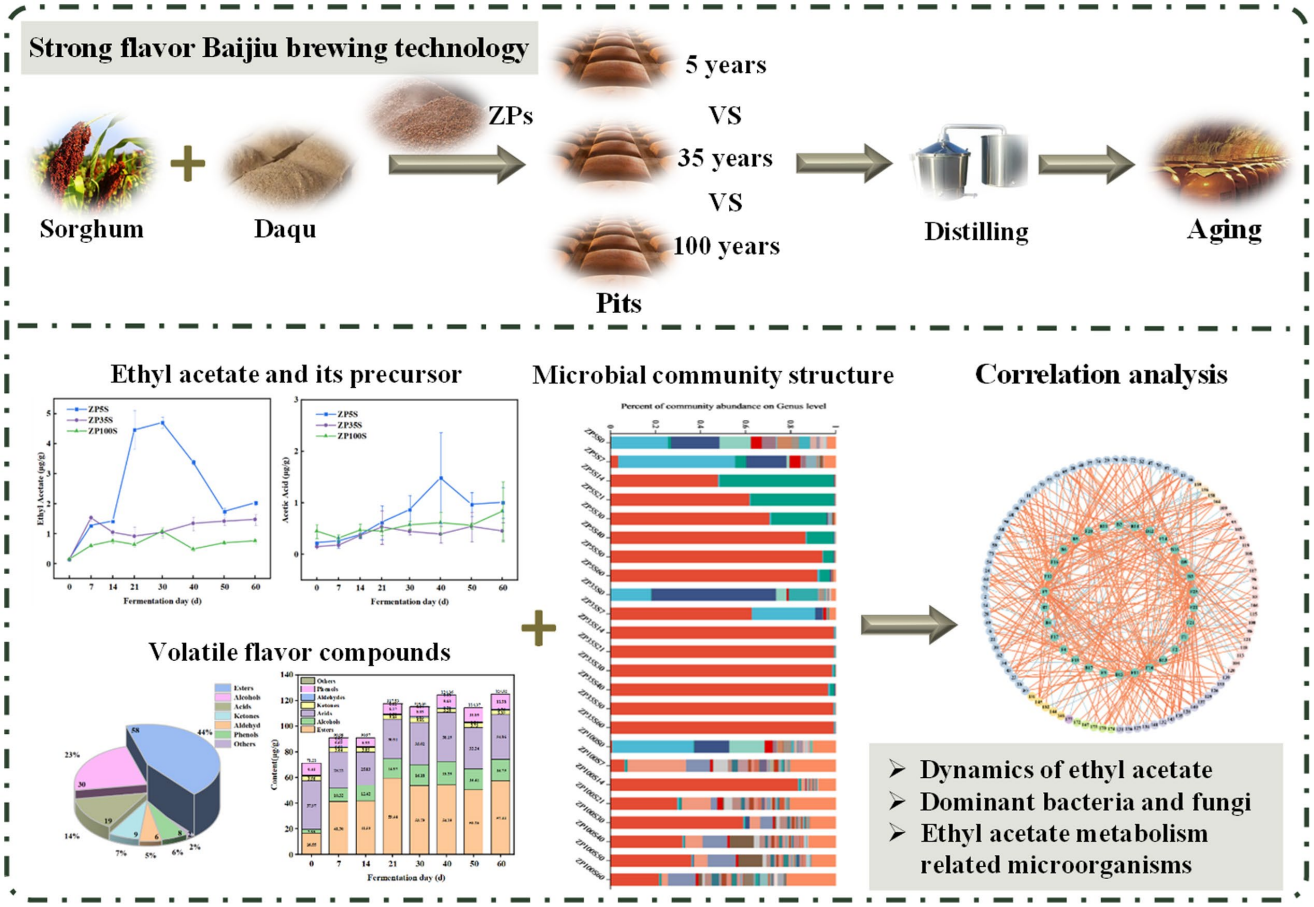
Effects of the pits of different ages on ethyl acetate metabolism during the fermentation of strong-flavor Baijiu.

Pits play a crucial role in Baijiu production, serving as essential equipment for strong-flavor Baijiu by providing a place for ZP (fermented grains) fermentation. In addition, the Baijiu brewing process is enhanced as the pit mud provides a rich source of microorganisms. However, the microbial community varies across pits of different ages, which may contribute to differences in Baijiu quality. To explore the effect of pits of different ages on Baijiu production, this study comprehensively analyzed the microbial community succession and ethyl acetate metabolism of ZPs from pits of different ages, using a combination of amplicon sequencing technology and HS-SPME–GC–MS. The key microorganisms involved in ethyl acetate metabolism and the environmental factors affecting the microbial activity were explored. This study provides a theoretical basis for controlling ethyl acetate levels and improving the quality of strong-flavor Baijiu (Scheme 1).

## 2 Materials and methods

### 2.1 Sample collection

All samples were collected from Luzhou Laojiao Group Co. Ltd., a strong-flavor Baijiu producer in Sichuan Province, China. ZPs from pits of different ages (5, 35, and 100 years) were collected at various fermentation time points (0, 7, 14, 21, 30, 40, 50, and 60 d), with a sampling depth of 50 cm from the ground. During sampling, the ZPs were collected from three locations along the diagonal of each pit and mixed uniformly as one sample. Each sample was divided into two parts and immediately placed into aseptic bags. One part was stored at −20°C for the analysis of physicochemical properties and volatile flavor components, while the other was stored at −80°C for amplicon sequencing. The samples obtained from the 5-year-old pit were marked as ZP5S0, ZP5S7, ZP5S14, ZP5S21, ZP5S30, ZP5S40, ZP5S50, and ZP5S60. The samples obtained from the 35-year-old pit were marked as ZP35S0, ZP35S7, ZP35S14, ZP35S21, ZP35S30, ZP35S40, ZP35S50, and ZP35S60. The samples obtained from the 100-year-old pit were marked as ZP100S0, ZP100S7, ZP100S14, ZP100S21, ZP100S30, ZP100S40, ZP100S50, and ZP100S60.

### 2.2 Analysis of the physicochemical properties

The pH and moisture contents were measured according to Xu’s research (Wang et al., 2017a). The acidity content was determined by acid-base titration (Guan et al., 2020). The starch and reducing sugar contents were tested following the methods of Ren (Ren et al., 2023).

### 2.3 Determination of the volatile flavor components

Sample pretreatment and headspace solid-phase microextraction were slightly modified based on previous research (Kong et al., 2014). In a 50 mL centrifuge tube, 5 g of the ZP was mixed with 20 mL of ultrapure water. The mixture was ultrasonicated at room temperature for 30 min and then centrifuged at 4°C (10,000 r/min, 30 min). The supernatant was collected and filtered through a 0.22 μm filter membrane. A 5 mL filtered solution was added to a 20 mL headspace solid-phase microextraction bottle containing 3 g of sodium chloride. Furthermore, 10 μL of 2-octanol (0.9427 g/L) was added as the internal standard. The samples were equilibrated at 50°C for 15 min and extracted for 40 min.

The GC–MS conditions were modified according to Duan’s research (Duan et al., 2023). The GC condition was as follows: an HP-INNOWAX column (60 m × 0.25 mm, 0.25 μm) was used, with high-purity helium (≥99.999%) as the carrier gas in the non-shunt mode, a flow rate of 1.0 mL/min, and an inlet temperature of 260°C. The temperature procedure was as follows: the temperature was initially maintained at 40°C for 5 min, then increased to 220°C at a rate of 5°C/min, followed by an increase to 250°C at a rate of 20°C/min, and finally was maintained at 250°C for 2.5 min. The MS conditions were as follows: the ionization mode was electron ionization (EI), with ion source and interface temperatures set to 230°C and 260°C, respectively. Full-scan acquisition was performed over a mass range of 35–450 amu to characterize the compounds. A qualitative analysis was performed by matching the NIST 20 standard library. According to the ratio of the peak area of the measured substance to the internal standard (2-octanol), the content of the volatile flavor components was calculated.

### 2.4 Amplicon sequencing

The ZPs were sent to Shanghai Meiji Biomedical Technology Co., Ltd. for the analysis of the microbial community structure using the PE300 platform (Illumina, San Diego, CA). The 16S rRNA V3-V4 region of bacteria and the ITS1-ITS2 region of fungi were extracted for amplicon sequencing. The sequencing data were analyzed using QIIME2 (Caporaso et al., 2010) and R packages. α diversity, β diversity, and microbial community composition analyses were provided by the Meiji Biomedical Online Platform.^1^

### 2.5 Statistical analysis

The data were processed and analyzed using SPSS (version 26.0). Graphs were created using OriginPro (version 2021). Redundancy analysis (RDA) and correspondence analysis (CA) were performed using Canoco (version 5.0). Visual diagrams were analyzed using Cytoscape (version 3.10.1).

## 3 Results

### 3.1 Dynamic changes in the physicochemical properties

**Figure 1:**
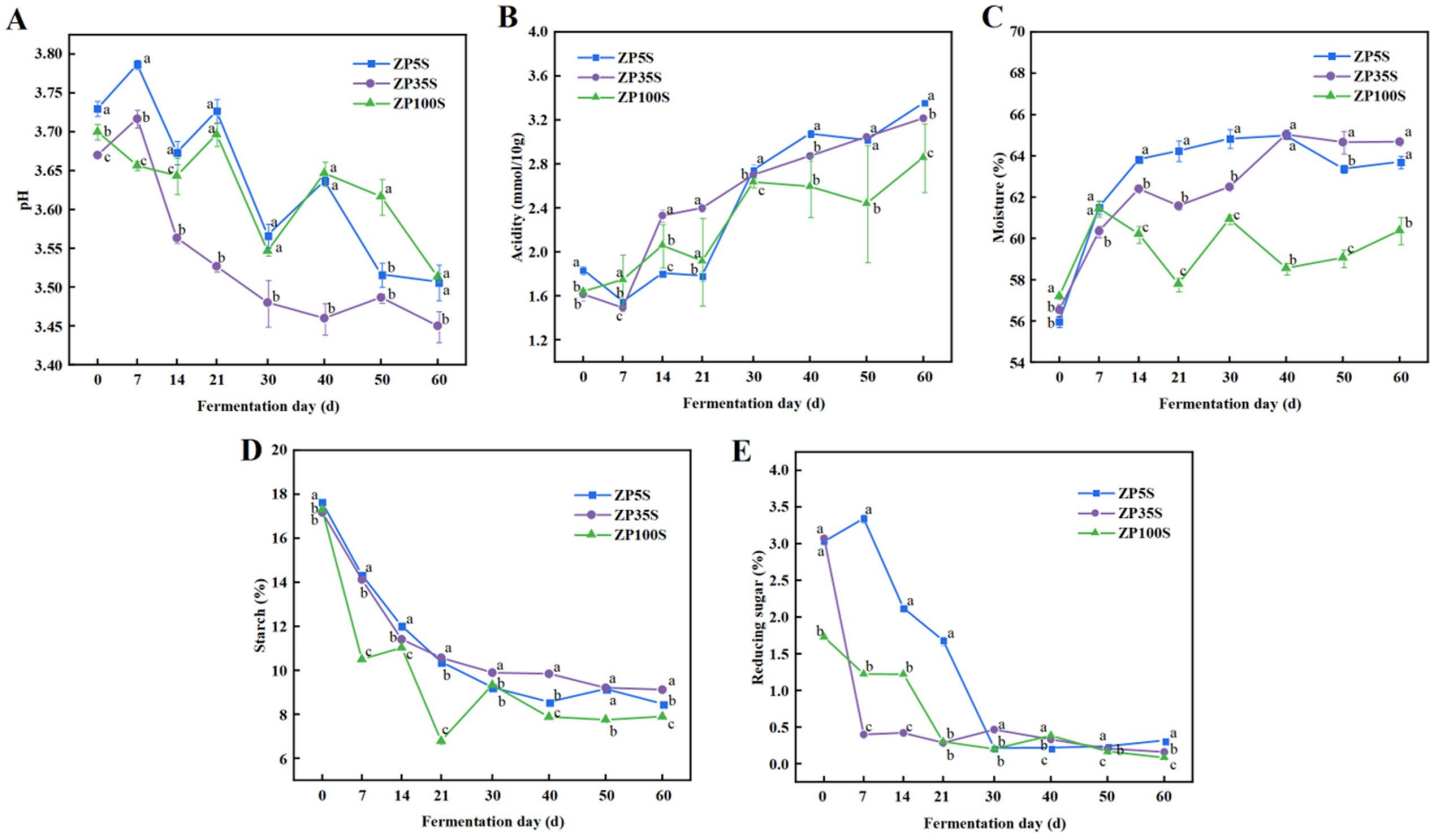
Dynamic changes in the physicochemical properties. **(A)** pH. **(B)** Acidity. **(C)** Moisture. **(D)** Starch. **(E)** Reducing sugars. Different letters represent statistically different values at *p* < 0.05.

During the entire fermentation process, the physicochemical properties of the ZPs changed significantly. As shown in Figure 1A, the pH level of the ZPs from the pits of different ages showed a downward trend, ranging from 3.4 to 3.8, with ZP35S showing the most rapid decrease. The changing trend in acidity was opposite to that of pH. As shown in Figure 1B, the acidity level of ZP5S, ZP35S, and ZP100S increased from 1.84, 1.59, and 1.64 mmol/10 g to 3.36, 3.21, and 2.68 mmol/10 g, respectively. The changes in moisture during the fermentation process are shown in Figure 1C. The moisture content of ZP5S, ZP35S, and ZP100S increased from 56.01, 56.64, and 57.21% to 63.70, 64.69, and 60.39%, respectively, with the most significant changes mainly occurring during the early stage of the fermentation. In addition, the starch content gradually decreased and eventually tended to stabilize as the fermentation proceeded, reaching approximately 8% at the end of the fermentation (Figure 1D). Furthermore, the reducing sugar content was also determined. As shown in Figure 1E, the change in reducing sugars mainly occurred within the first 30 d of the fermentation. It presented a general downward trend and tended to stabilize after 30 d.

### 3.2 Volatile flavor components

**Figure 2:**
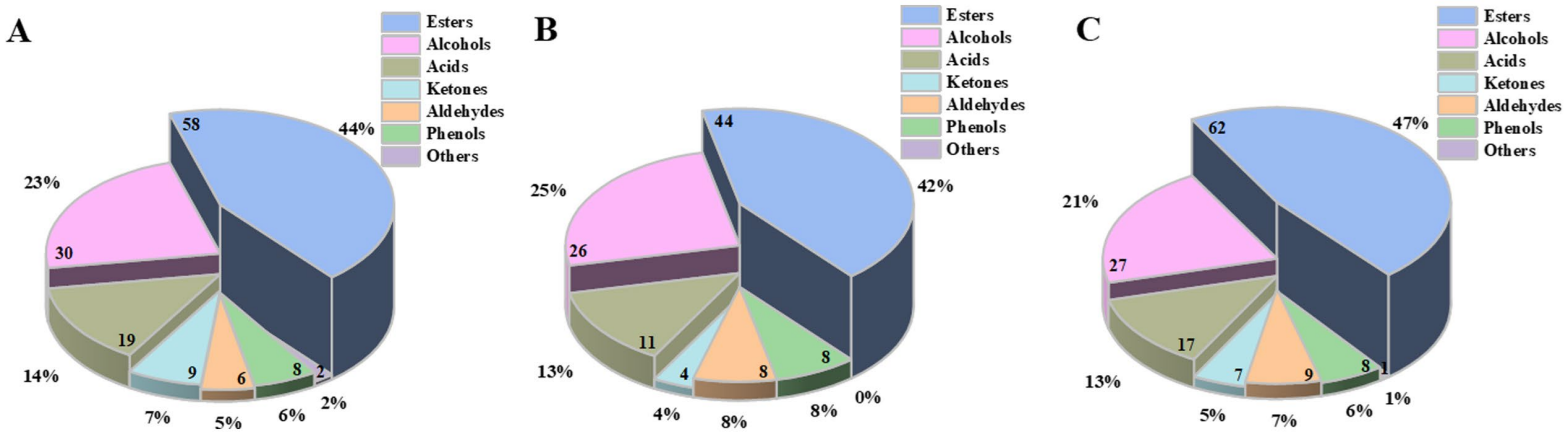
Percentage of the volatile flavor components in the ZPs. **(A)** ZP5S. **(B)** ZP35S. **(C)** ZP100S. Numbers on the pie chart represent the quantity of the volatile flavor components in each category.

A total of 177 types of volatile flavor components were identified from the pits of different ages of the ZPs, including 80 esters, 42 alcohols, 21 acids, 10 ketones, 11 aldehydes, 10 phenols, and 3 others (Supplementary Table S1). As shown in Figure 2 and Supplementary Table S2, the volatile flavor components showed significant differences among the three kinds of ZPs. Esters, alcohols, and acids were the main volatile flavor components during the fermentation of strong-flavor Baijiu, accounting for over 80% of the total quantity.

**Figure 3:**
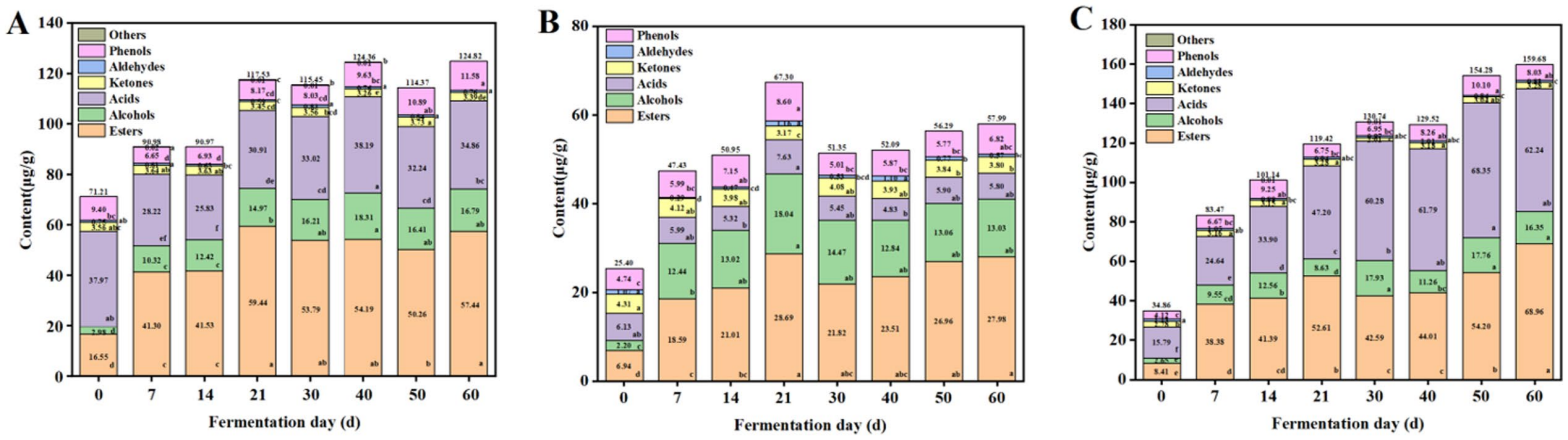
Variations in the volatile flavor compounds in the ZPs. **(A)** ZP5S. **(B)** ZP35S. **(C)** ZP100S. Different letters represent statistically different values at *p* < 0.05.

The content changes of the volatile flavor compounds were also analyzed. As shown in Figure 3A, during the fermentation, the content of the esters in ZP35S continuously accumulated, reaching a maximum value of 59.44 μg/g on the 21st day. The acids showed a downward trend during the first 14 d, followed by an increase from the 14th to the 40th day. The alcohols continued to accumulate, reaching a maximum value of 18.29 μg/g on the 40th day. The phenols initially decreased and then increased. The aldehydes and ketones fluctuated with low contents. The content changes of the volatile flavor compounds in ZP35S and ZP100S are shown in Figures 3B,C, where the volatile flavor compounds exhibited a similar trend to that of ZP5S. The formation of the volatile flavor compounds mainly occurred within the first 21 d of the fermentation. Compared to ZP5S and ZP35S, the content of the esters, alcohols, and acids, as well as the total content of the volatile flavor compounds, was higher in ZP100S.

**Figure 4:**
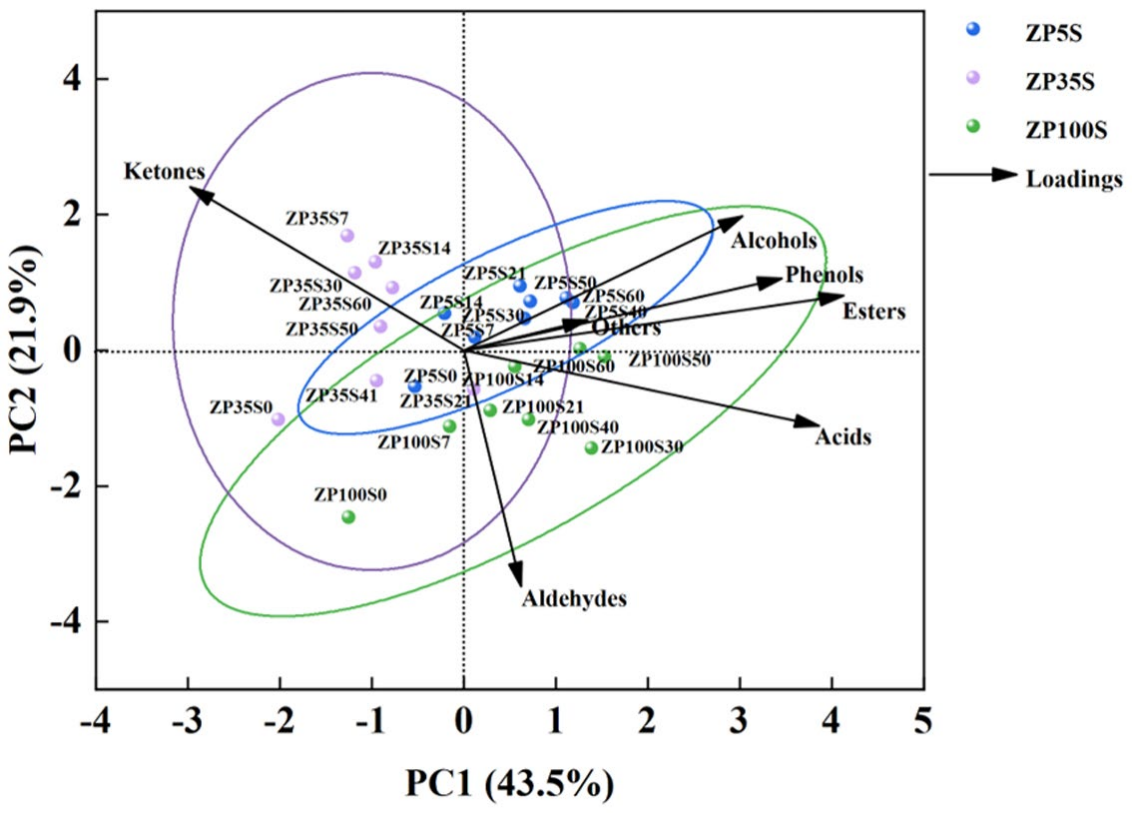
PCA of the volatile flavor compounds in the ZPs.

The content of the esters, alcohols, acids, ketones, aldehydes, phenols, and others were used as independent variables for principal component analysis (PCA), and the results are shown in Figure 4. The total variance contribution rate of PC1 and PC2 was 65.4%, which reflected the general characteristics of the volatile flavor compounds. The ZP5S samples were mainly distributed in the first quadrant, reflecting the contribution of the esters, alcohols, and phenols, indicating that these volatile flavor compounds played a certain role in the formation of flavor in ZP5S. The ZP35S samples were mainly distributed in the second quadrant, reflecting the influence of the ketones, which contributed to the flavor of ZP35S. The ZP100S samples were mainly distributed in the fourth quadrant, reflecting the contribution of the acids and aldehydes. The samples were scattered according to the different ages of the pits, indicating that pit age had an effect on the difference in the volatile flavor compounds.

**Figure 5:**
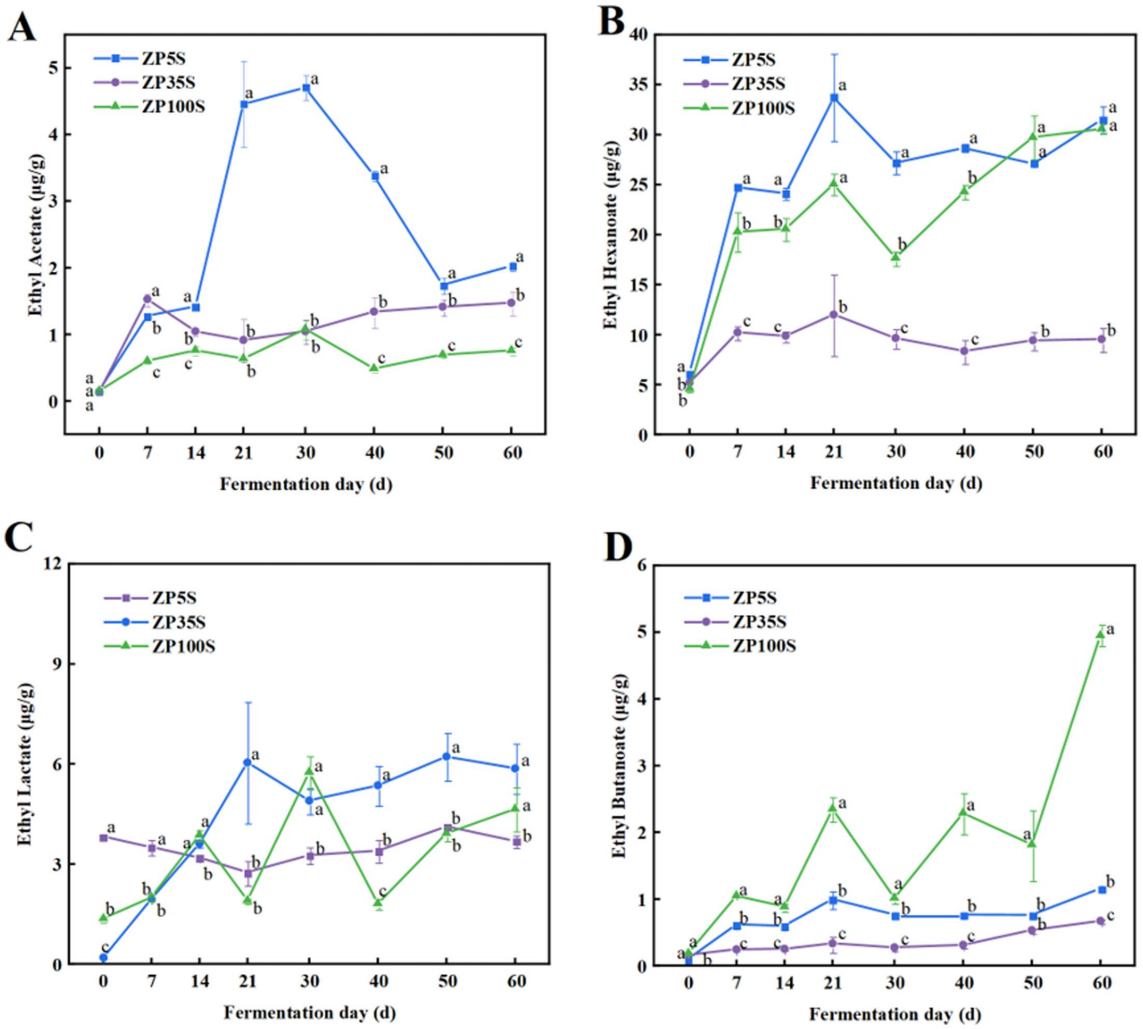
Dynamic changes in the essential esters. **(A)** Ethyl acetate. **(B)** Ethyl hexanoate. **(C)** Ethyl lactate. **(D)** Ethyl butanoate. Different letters represent statistically different values at *p* < 0.05.

Ethyl hexanoate, ethyl acetate, ethyl butyrate, and ethyl lactate are the four most important ethyl esters in strong-flavor Baijiu (Guan et al., 2023). As shown in Figure 5, ethyl acetate increased to varying degrees, with the highest content observed in ZP5S. Ethyl hexanoate gradually increased during the first 21 d of the fermentation, showing similar content across the three ZPs at the end of the fermentation As the fermentation progressed, the content of ethyl lactate in ZP35S and ZP100S gradually increased. In addition, the content of ethyl butyrate in ZP100S was significantly higher than in ZP5S and ZP35S.

**Figure 6:**
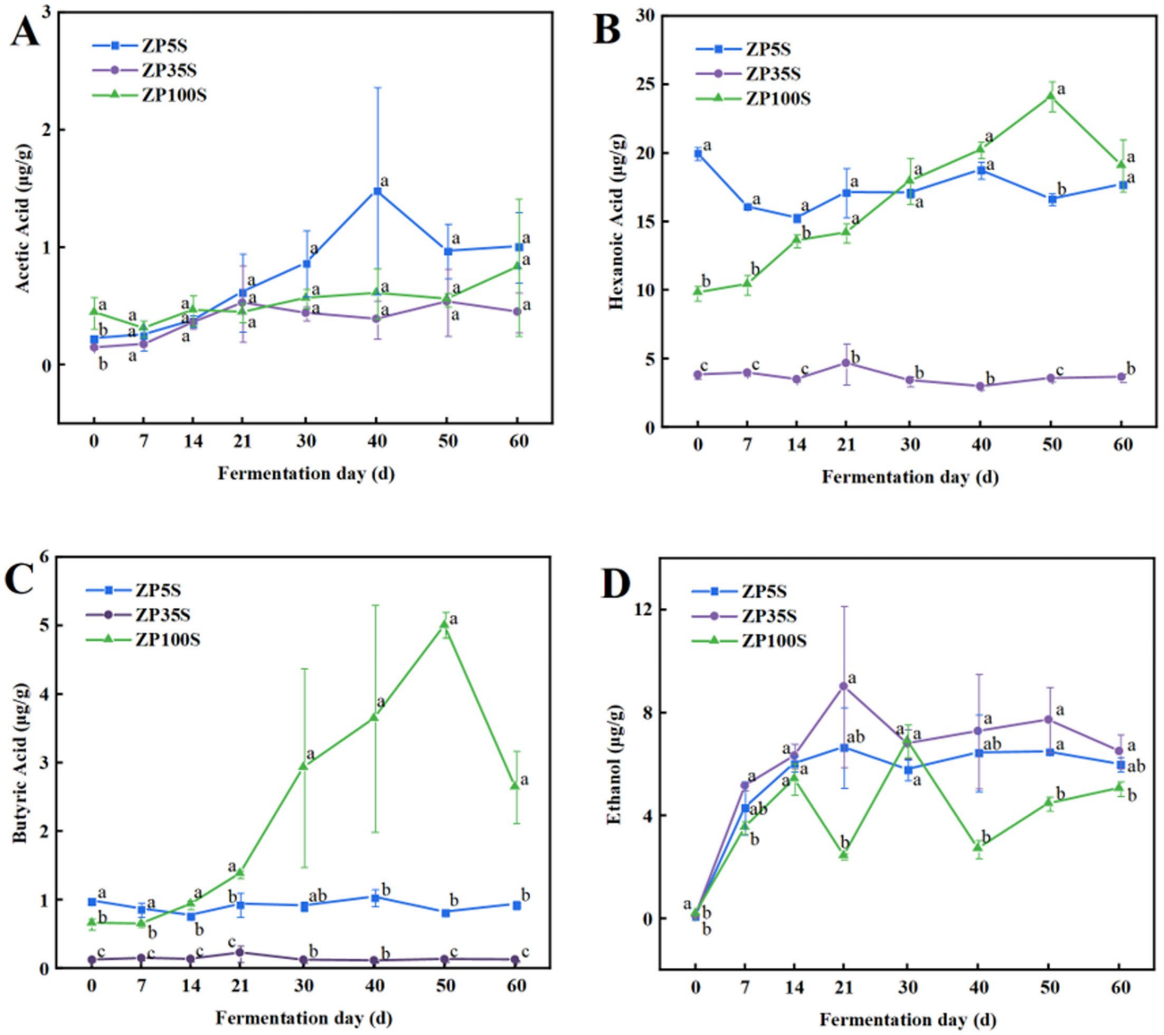
Dynamic changes in ethanol and essential acids. **(A)** Acetic acid. **(B)** Hexanoic acid. **(C)** Butyric acid. **(D)** Ethanol. Different letters represent statistically different values at *p* < 0.05.

Acetic acid, hexanoic acid, butyric acid, and ethanol are not only important components with strong aroma and taste but also essential substances involved in the formation of the main esters in strong-flavor Baijiu. As shown in Figure 6, acetic acid in ZP5S showed an overall increasing trend and reached a maximum value of 1.48 μg/g on the 40th day. At the end of the fermentation, acetic acid presented the highest content in ZP5S, followed by ZP100S and ZP35S. Hexanoic acid in ZP100S continuously accumulated and reached a maximum value of 24.11 μg/g on the 50th day of the fermentation. Overall, the content of hexanoic acid in ZP35S was significantly lower than that in ZP100S and ZP5S. Butyric acid in ZP5S and ZP35S exhibited relatively low levels throughout the fermentation process. On the contrary, butyric acid gradually accumulated in ZP100S and reached a maximum value of 5.01 μg/g on the 50th day. The ethanol content in the three kinds of ZPs exhibited a similar increasing trend.

### 3.3 Microbial diversity analysis

#### 3.3.1 α diversity

After splicing and filtering the data, 1,145,923 and 2,331,631 effective sequences were obtained from the bacteria and fungi, respectively. The coverage of the high-quality sequences for each sample was higher than 99%, indicating that the sequencing data accurately reflected the microbial community (Du et al., 2020). The richness of the microbial community structure during the fermentation of the ZPs was analyzed, and the diversity indexes of the bacteria and fungi were calculated based on a 97% similarity level. As shown in the results of bacterial diversity in Supplementary Table S3, the ACE, Chao, and Shannon index values of ZP100S were higher than those of ZP5S and ZP35S throughout the fermentation process. In addition, the Simpson’s diversity index value of ZP100S was the lowest. The results demonstrated that the bacteria community in ZP100S was the richest. The diversity of the fungi was also analyzed (Supplementary Table S4). ZP35S exhibited the highest ACE, Chao, and Shannon index values, along with the lowest Simpson’s diversity index value, suggesting good abundance and diversity among the three ZPs.

#### 3.3.2 β diversity

**Figure 7:**
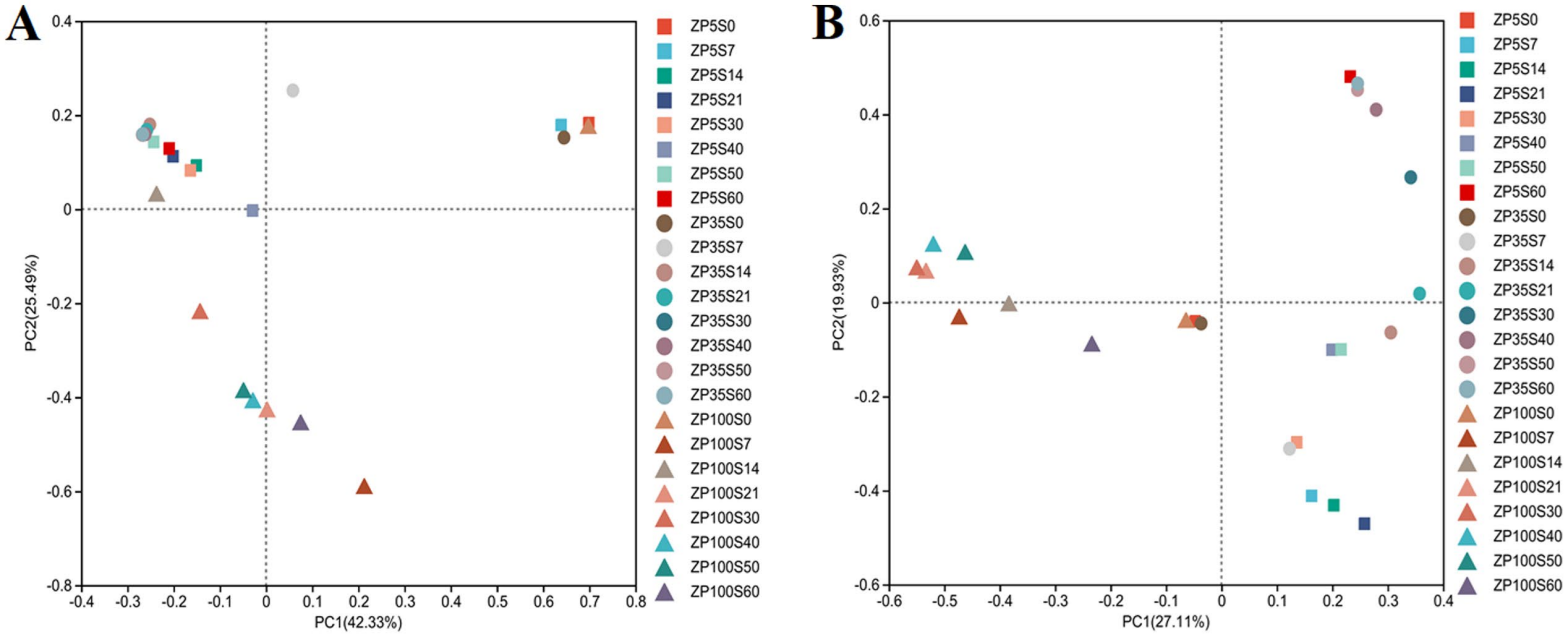
PCoA of the microbial community structure during the fermentation of the ZPs. **(A)** Bacteria. **(B)** Fungi.

Based on the Bray–Curtis distance coefficient, Principal Coordinates Analysis (PCoA) was conducted to assess the microbial community structure. As shown in Figure 7A, the explanation rates of principal component 1 (PC1) and principal component 2 (PC2) for the variation in the bacterial community structure were 42.33 and 25.49%, respectively, which sufficiently reflected the differences in the bacterial community structure among the samples. The samples of ZP5S0, ZP35S0, and ZP100S0 were close to each other and overlapped, indicating a similar bacterial community structure at the beginning of the fermentation. The samples of ZP5S and ZP35S from days 14 to 60 were grouped together. In contrast, the samples of ZP100S from days 21 to 60 were separated from the others, indicating that the succession of the bacterial community structure in ZP100S differed significantly from that in ZP5S and ZP35S. In addition, the succession of the fungal community structure was also explored. As shown in Figure 7B, the fungi exhibited a similar community structure at the beginning of the fermentation. As the fermentation progressed, the succession of the fungi showed obvious differences among the three ZPs.

### 3.4 Microbial community structure

**Figure 8:**
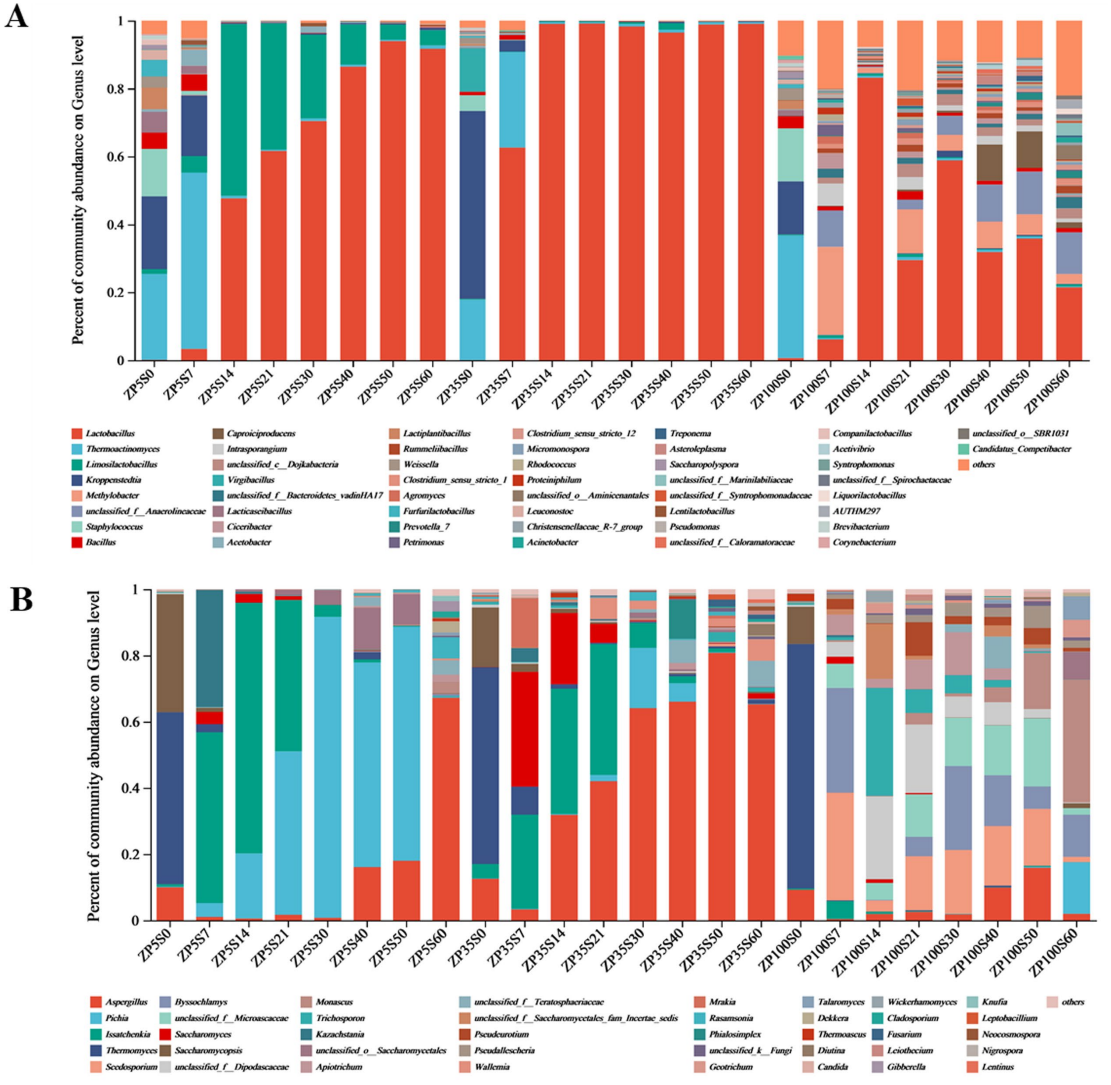
Microbial community structure of the ZPs during the fermentation of Baijiu (genus level). **(A)** Bacteria. **(B)** Fungi.

A total of 42 bacterial phyla and 690 bacterial genera were identified from all samples. The microorganisms with a relative abundance greater than 1% in a sample were defined as dominant, while the rest were classified as others. According to this criterion, 11 dominant phyla were found in the three ZPs (Supplementary Figure S1A). Throughout the fermentation process, Firmicutes was the dominant phylum in ZP5S (89.93–99.66%) and ZP35S (96.12–99.93%). The dominant bacterial phyla in ZP100S were more abundant. At the genus level, 51 bacterial genera with a relative abundance greater than 1% were detected in ZP5S, ZP35S, and ZP100S (Figure 8A). As the fermentation progressed, significant differences were observed in the composition and succession of the bacteria in ZP5S, ZP35S, and ZP100S. At the beginning of the fermentation, the number of dominant bacterial genera in ZP5S, ZP35S, and ZP100S were 13, 6, and 14, respectively. *Thermoactinomyces*, *Kroppenstedtia*, *Staphylococcus*, *Bacillus,* and *Weissella* were the common dominant bacteria genera in the three types of ZPs. On the 7th day, the relative abundance of *Thermoactinomyces* in ZP5S and ZP35S was higher than on the 0th day. The relative abundance of *Kroppenstedtia* showed a downward trend in all three kinds of ZPs. Furthermore, the relative abundance of *Lactobacillus* increased. On the 14th day, the abundance of *Thermoactinomyces* and *Kroppenstedtia* in all three kinds of ZPs evidently decreased, and they remained relatively unchanged thereafter. *Lactobacillus* in ZP5S gradually increased and became the dominant genus. In ZP35S, *Lactobacillus* evidently increased compared to the 7th day, gaining a clear advantage over all other bacteria genera. As the fermentation progressed, the relative abundance of *Lactobacillus* fluctuated in ZP100S. In addition, throughout the fermentation, other genera, including *Methylobact*, *unclassified_f_Anaerolineaceae*, *unclassified_c_Dojkabacteria*, *Ciceribacter*, *Rummeliibacillus*, *Agromyces*, *Petrimonas*, *Micromonospora*, *Rhodococcus*, and *Proteiniphilum,* were also detected, with their relative abundance gradually decreasing.

A total of nine fungal phyla and 155 fungal genera were identified from all samples. As shown in Supplementary Figure S1B, three fungal phyla—Ascomycota, Basidiomycota, and unclassified_k_Fungi—were considered the dominant phyla, each with a relative abundance of more than 1%. Among them, Ascomycota was the most dominant phylum during the fermentation of strong-flavor Baijiu, with relative abundance values ranging from 95.75 to 99.99% in ZP5S, 83.37 to 98.73% in ZP35S, and 64.20 to 99.22% in ZP100S. At the genus level, 40 fungal genera with a relative abundance greater than 1% were detected in the three kinds of ZPs (Figure 8B). At the beginning of the fermentation, the number of dominant fungal genera in ZP5S, ZP35S, and ZP100S were 3, 4, and 4, respectively. On the 7th day of the fermentation, the relative abundance of *Aspergillus*, *Thermomyces,* and *Saccharomycopsis* decreased, while *Issatchenkia*, *Kazachstania*, *Pichia*, *Saccharomyces*, *Scedosporium,* and *Byssochlamys* increased and became the dominant genera. From days 14 to 50, the relative abundance of *Issatchenkia* and *Saccharomyces* in ZP5S continued to decrease to low levels, while *Pichia* gradually increased and reached 90.89% on the 30th day. *Issatchenkia* and *Saccharomyces* in ZP35S showed a similar trend to that in ZP5S. *Aspergillus* in ZP35S continuously increased and reached 80.76% on the 50th day. The dominant fungal genera in ZP100S were more abundant in composition and more complicated in variation.

### 3.5 Analysis of the differential microorganisms

**Figure 9:**
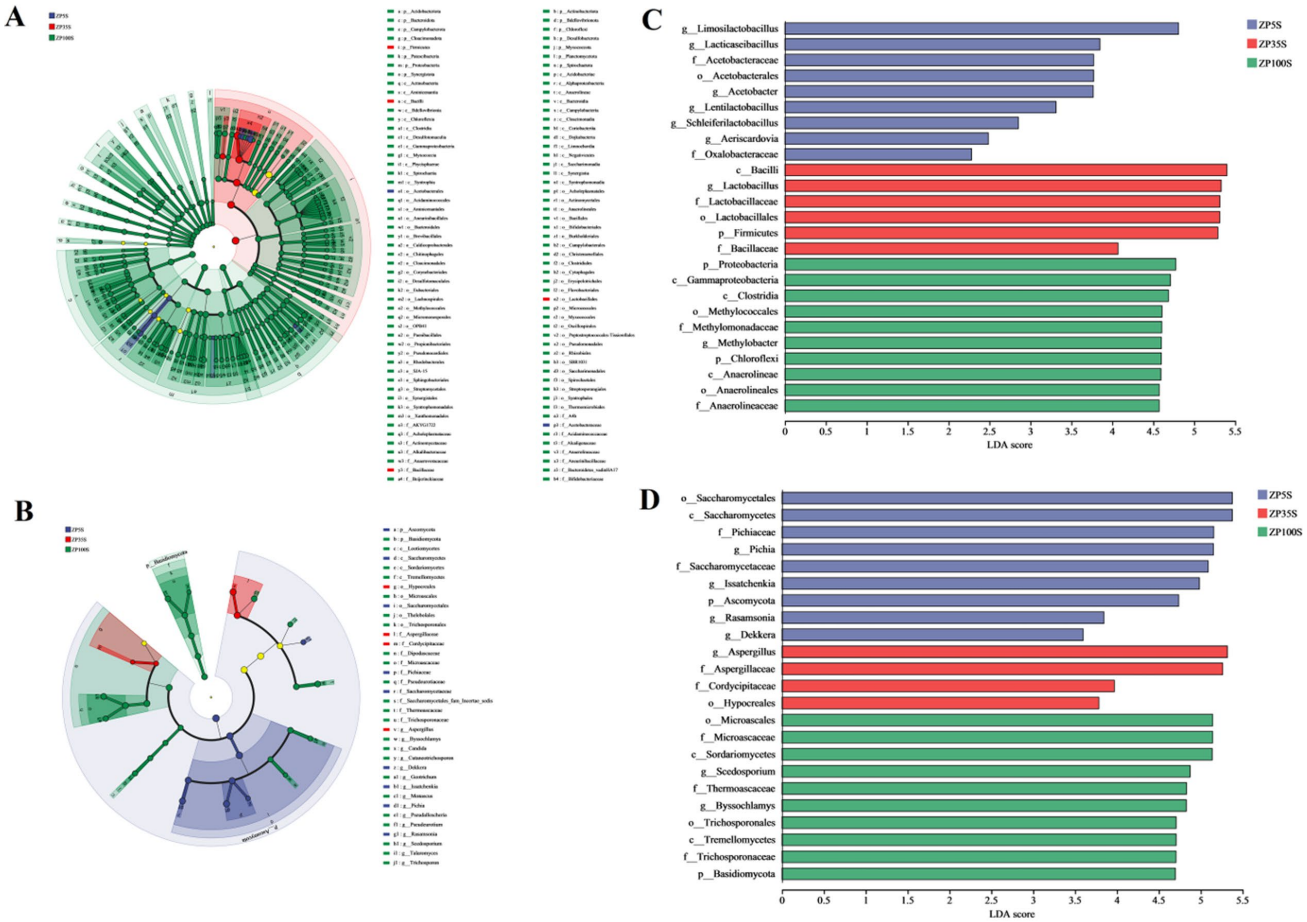
Analysis of the differential microorganisms in ZP5S, ZP35S, and ZP100S. **(A)** LEfSe multilevel species hierarchical tree of the bacteria. **(B)** LEfSe multilevel species hierarchical tree of the fungi. **(C)** LDA score plot of the bacteria. **(D)** LDA score plot of the fungi.

To further investigate the differences among ZP5S, ZP35S, and ZP100S, the LDA Effect Size (LEfSe) was performed Figures 9A,B. As shown in Figures 9C,D, six bacterial genera (*Limosilactobacillus*, *Acetobacter*, *Lacticaseibacillus*, *Lentilactobacillus*, *Schleiferilactobacillus*, and *Aeriscardovia*) and four fungal genera (*Pichia*, *Issatchenkia*, *Rasamsonia*, and *Dekkera*) with significant differences were detected in ZP5S. One bacterial genus (*Lactobacillus*) and one fungal genus (*Aspergillus*) with significant differences were detected in ZP35S. One bacterial genus (*Methylobacter*) and two fungal genera (*Scedosporium and Byssochlamys*) with significant differences were detected in ZP100S.

### 3.6 Correlation analysis of the microorganisms with the physicochemical properties and volatile flavor components

**Figure 10:**
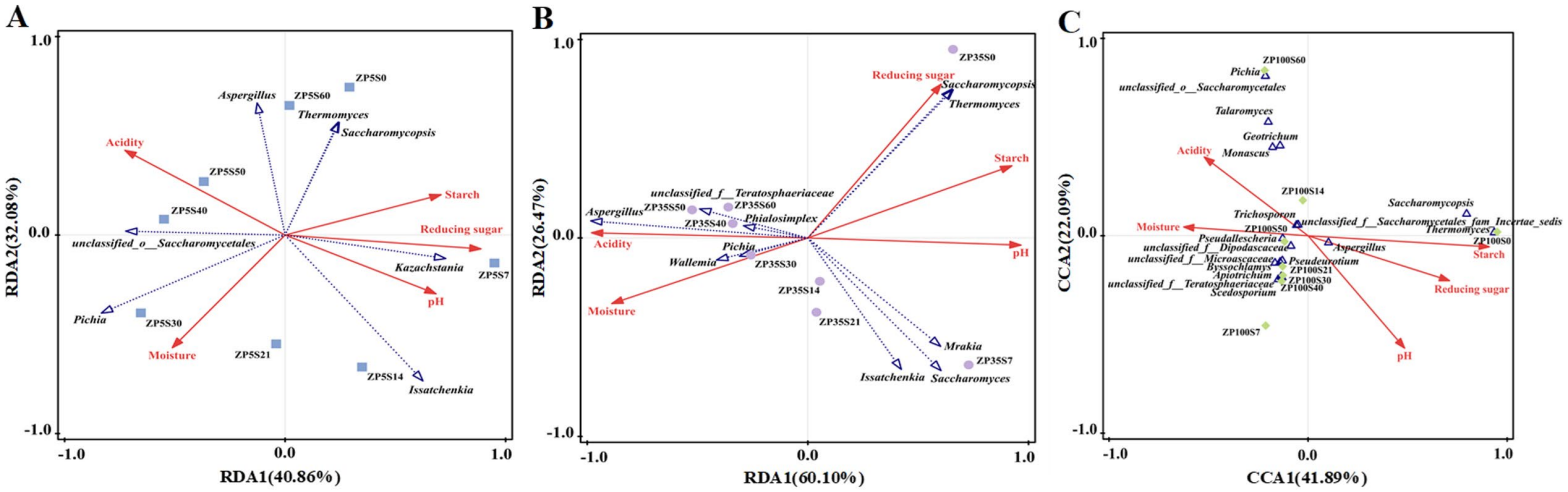
RDA or CA of the dominant fungi genera and physicochemical properties. **(A)** ZP5S. **(B)** ZP35S. **(C)** ZP100S.

The potential relationships between the dominant genera and main physicochemical properties were analyzed using RDA and CA. As shown in Figure 10, the first two axes explained 72.94, 86.57, and 63.98% of the variation in the fungal community differentiation of ZP5S, ZP35S, and ZP100S, respectively, suggesting certain correlations between the microbial community and physicochemical properties. *Pichia* was positively correlated with acidity and moisture and negatively correlated with starch and reducing sugars. *Saccharomycopsis* and *Thermomyces* showed a positive correlation with starch and reducing sugars. The correlation between the bacteria community and physicochemical properties is shown in Supplementary Figure S2. *Lactobacillus* was significantly positively correlated with acidity. *Thermoactinomyces*, *Staphylococcus*, *Kroppenstedtia*, and *Bacillus* were positively correlated with starch, reducing sugars, and pH and negatively correlated with acidity and moisture.

**Figure 11:**
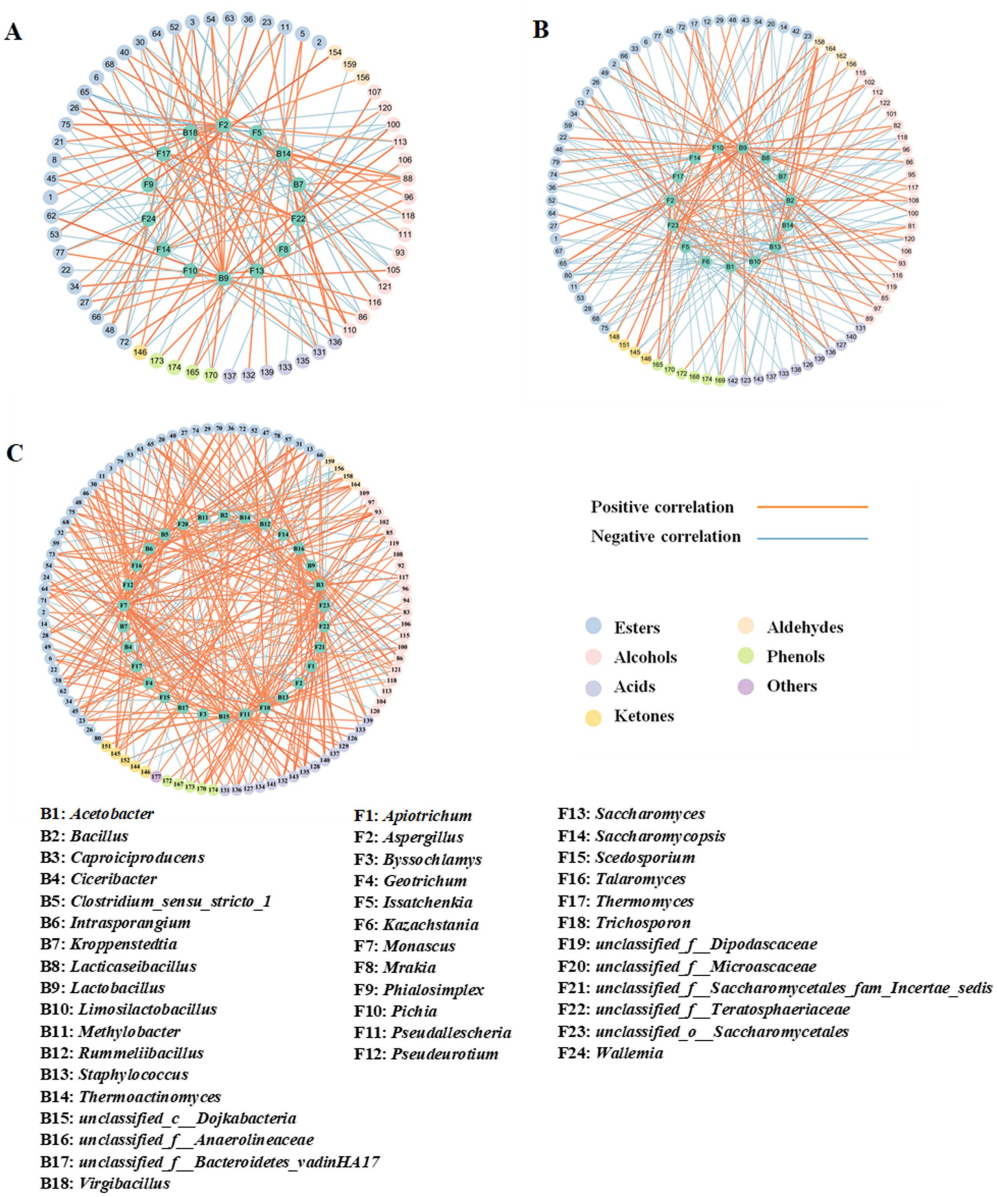
Spearman correlation analysis of the dominant genera and volatile flavor components. **(A)** ZP5S. **(B)** ZP35S. **(C)** ZP100S. The different volatile flavor compounds are represented by the outer circle, while the various dominant genera are represented by the inside circle. The absolute value of the Spearman rank correlation coefficient was greater than 0.7. The orange line represents a positive correlation, and the blue line represents a negative correlation. The thicker the line, the stronger the correlation.

Co-occurrence network analysis was performed based on Spearman correlation analysis between the dominant microbial genera (relative abundance >1%) and volatile flavor components. A correlation coefficient with an absolute value greater than 0.7 was selected for visual analysis. As shown in Figure 11A and Supplementary Table S5, in ZP5S, 15 dominant genera exhibited a strong correlation (|*R*| > 0.7) with at least one of 88 volatile flavor components, including 44 esters, 22 alcohols, 12 acids, four ketones, four aldehydes, and six phenols. In ZP35S, 14 dominant genera showed a strong correlation (|*R*| > 0.7) with at least one of 60 volatile flavor components, including 30 esters, 15 alcohols, seven acids, one ketone, three aldehydes, and four phenols (Figure 11B; Supplementary Table S6). Compared to ZP5S and ZP35S, the correlation between the microorganisms and volatile flavor components was more complex in ZP100S. A total of 30 dominant genera showed a strong correlation (|*R*| > 0.7) with at least one of 97 volatile flavor components, including 46 esters, 21 alcohols, 15 acids, five ketones, four aldehydes, five phenols, and one other (Figure 11C; Supplementary Table S7).

Ethyl hexanoate, ethyl lactate, ethyl butyrate, and ethyl acetate are important flavor components in strong-flavor Baijiu, produced through an esterification reaction using ethanol and acids as precursors (Xu Y. Q. et al., 2022). As shown in Figure 11A, in ZP5S, ethyl acetate was positively correlated with *Pichia* (*R* = 0.714, *p* < 0.05) and negatively correlated with *Saccharomycopsis* (*R* = −0.714, *p* < 0.05) and *Staphylococcus* (*R* = −0.764, *p* < 0.05). Ethyl lactate showed negative correlations with *Issatchenkia* (*R* = −0.738, *p* < 0.05) and *Limosilactobacillus* (*R* = −0.905, *p* < 0.01). Acetic acid was positively correlated with *Lactobacillus* (*R* = 0.905, *p* < 0.01) and negatively correlated with *Staphylococcus* (*R* = −0.764, *p* < 0.05), *Bacillus* (*R* = −0.905, *p* < 0.01), and *Acetobacter* (*R* = −0.714, *p* < 0.05).

In ZP35S, *Issatchenkia* (*R* = 0.738, *p* < 0.05) and *Saccharomyces* (*R* = 0.881, *p* < 0.01) were positively correlated with ethyl hexanoate. Ethyl lactate was positively correlated with *Lactobacillus* (*R* = 0.714, *p* < 0.05) and *Aspergillus* (*R* = 0.810, *p* < 0.05) and negatively correlated with *Virgibacillus* (*R* = −0.709, *p* < 0.05), *Thermomyces* (*R* = −0.714, *p* < 0.05), and *Saccharomycopsis* (*R* = −0.762, *p* < 0.05). Ethyl butyrate showed positive correlations with *Lactobacillus* (*R* = 0.738, *p* < 0.05) and *Aspergillus* (*R* = 0.810, *p* < 0.05) and negative correlations with *Thermoactinomyces* (*R* = −0.714, *p* < 0.05), *Virgibacillus* (*R* = −0.709, *p* < 0.05), and *Saccharomycopsis* (*R* = −0.714, *p* < 0.05). Acetic acid was positively correlated with *Lactobacillus* (*R* = 0.738, *p* < 0.05) and *Aspergillus* (*R* = 0.762, *p* < 0.05) and negatively correlated with *Thermoactinomyces* (*R* = −0.714, *p* < 0.05), *Virgibacillus* (*R* = −0.791, *p* < 0.05), *Thermomyces* (*R* = −0.786, *p* < 0.05), and *Saccharomycopsis* (*R* = −0.786, *p* < 0.05). In addition, *Saccharomyces* (*R* = 0.810, *p* < 0.05) showed a positive correlation with butyric acid.

In ZP100S, six genera including *unclassified_f_Anaerolineaceae* (*R* = 0.714, *p* < 0.05), *Caproiciproducens* (*R* = 0.714, *p* < 0.05), *Clostridium_sensu_stricto_1* (*R* = 0.857, *p* < 0.01), *Monascus* (*R* = 0.881, *p* < 0.01), *Geotrichum* (*R* = 0.719, *p* < 0.05), and *unclassified_o_Saccharomycetales* (*R* = 0.833, *p* < 0.05) were positively correlated with ethyl hexanoate. Ethyl acetate was positively correlated with *Lactobacillus* (*R* = 0.738, *p* < 0.05) and negatively correlated with *Thermomyces* (*R* = −0.934, *p* < 0.01). Ethyl lactate showed a positive correlation with *Pichia* (*R* = 0.905, *p* < 0.01) and a negative correlation with *Thermomyces* (*R* = −0.970, *p* < 0.01). A total of six genera—*unclassified_c_Dojkabacteria* (*R* = 0.714, *p* < 0.05), *unclassified_f_Bacteroidetes_vadinHA17* (*R* = 0.762, *p* < 0.05), *Clostridium_sensu_stricto_1* (*R* = 0.881, *p* < 0.01), *Monascus* (*R* = 0.881, *p* < 0.01), *Talaromyces* (*R* = 0.881, *p* < 0.01), and *Rummeliibacillus* (*R* = 0.929, *p* < 0.01)—were positively correlated with ethyl butyrate. *Pseudallescheria* (*R* = 0.833, *p* < 0.05), *Monascus* (*R* = 0.881, *p* < 0.01), and *Caproiciproducens* (*R* = 1, *p* < 0.01) were positively correlated with hexanoic acid. *Caproiciproducens* (*R* = 0.786, *p* < 0.05) and *Monascus* (*R* = 0.738, *p* < 0.05) showed positive correlations with acetic acid. *Unclassified_f_Microascaceae* (*R* = 0.762, *p* < 0.05), *Monascus* (*R* = 0.738, *p* < 0.05), *unclassified_f_Teratosphaeriacea* (*R* = 0.819, *p* < 0.05), *Pseudallescheria* (*R* = 0.881, *p* < 0.05), and *Caproiciproducens* (*R* = 0.952, *p* < 0.01) were positively correlated with butyric acid.

**Figure 12:**
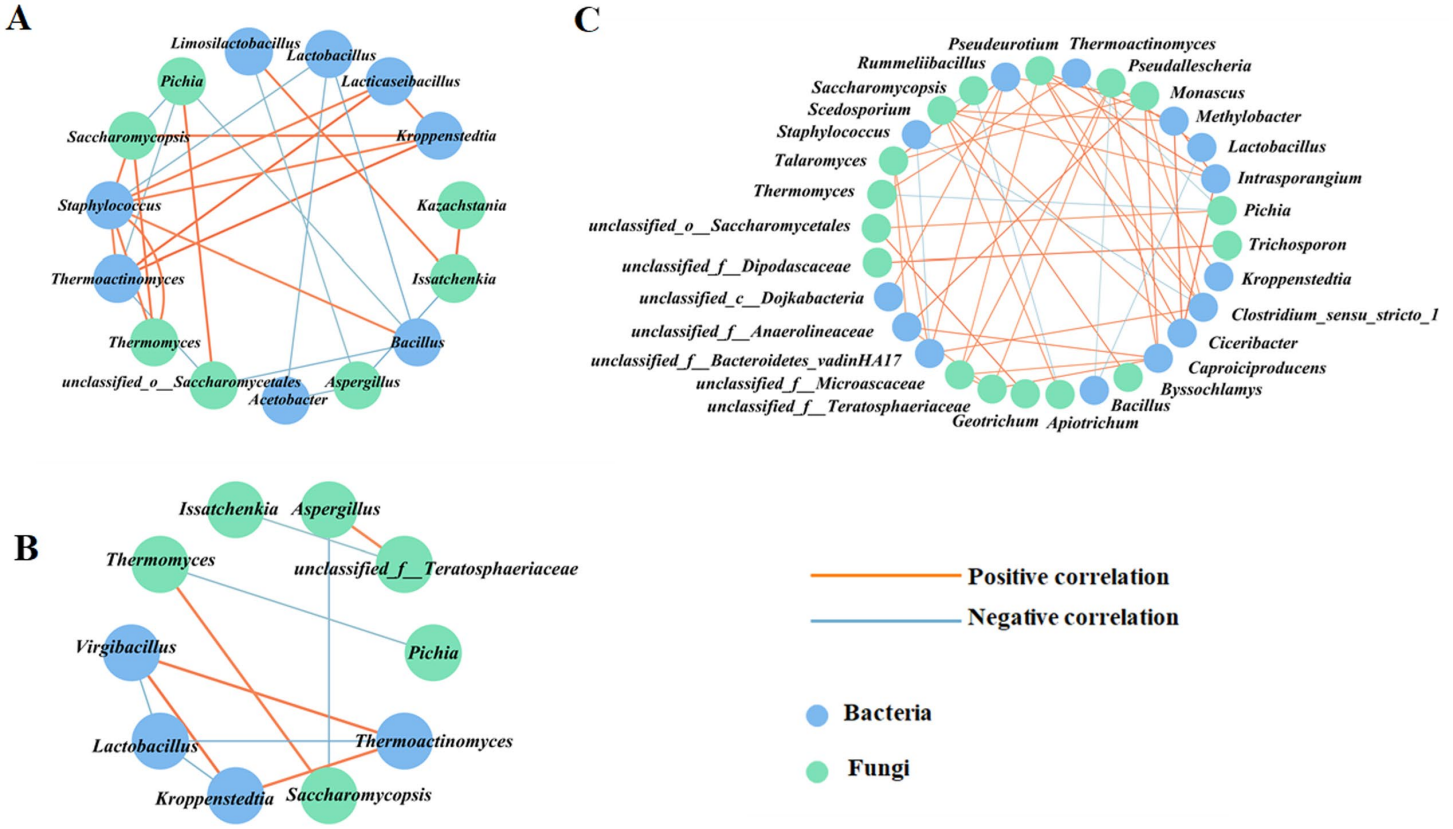
Correlation network analysis of the dominant genera. **(A)** ZP5S. **(B)** ZP35S. **(C)** ZP100S. The circle represents the dominant genera. The absolute value of the Spearman rank correlation coefficient was greater than 0.7. The orange line represents a positive correlation, and the blue line represents a negative correlation. The thicker the line, the stronger the correlation.

Based on the Spearman correlation analysis, co-occurrence network analysis of the microbial interactions at the genus level was performed using a correlation coefficient with an absolute value greater than 0.7. As shown in Figures 12A,B; Supplementary Tables S8, S9, a total of 15 nodes and 26 edges were observed in ZP5S, while 10 nodes and 11 edges were observed in ZP35S. The interaction of the microorganisms was more complex in ZP100S (Figure 12C; Supplementary Table S10). A total of 31 effective connection nodes and 47 edges were observed, with most showing a positive correlation, except for eight negative correlations. *Lactobacillus* was positively correlated with *Pseudallescheria* and negatively correlated with *Bacillus. Monascus* showed a positive correlation with *unclassified_f_Anaerolineaceae*, *Caproiciproducens*, *Rummeliibacillus,* and *Clostridium_sensu_stricto_1. Pichia* was negatively correlated with *Thermoactinomyces* and *Thermomyces* and positively correlated with *unclassified_o_Saccharomycetales*. These results indicate that the microbial communities in ZPs can coordinate and regulate each other.

## 4 Discussion

The amplicon sequencing results showed that Firmicutes and Ascomycota were the most dominant phyla and *Lactobacillus* was the dominant genus in the ZPs. These findings are consistent with those of previous studies (Wang et al., 2017b; Zhao et al., 2022). At the beginning of the fermentation, the microbial community composition was similar across all three kinds of ZPs. *Thermoactinomyces*, *Kroppenstedtia*, *Staphylococcus*, *Bacillus,* and *Weissella* were the dominant bacterial genera, while *Aspergillus*, *Thermomyces*, and *Saccharomycopsis* were the dominant fungal genera in all three kinds of ZPs. The results of the PCoA also showed that the bacterial and fungal communities of ZP5S, ZP35S, and ZP100S on the 0th day were similar or coincident. As the fermentation progressed, the composition and succession of the microbial community in ZP5S, ZP35S, and ZP100S differed, which was a result of the microorganisms adapting to the variation in the fermentation environment. Relative research has demonstrated that pits are an essential factor affecting the quality of Baijiu by regulating the succession of microorganisms during the fermentation of ZPs (Xu S. S. et al., 2022). Microorganisms in pit mud can participate in the fermentation of ZPs and exhibit different effects on the bacterial and fungal communities of ZPs (Qian et al., 2021; Wang et al., 2017b). Overall, the microbial composition of ZP100S was more abundant than that of ZP5S and ZP35S. The older the pit, the more abundant the functional microorganisms (Zhang et al., 2020). It was speculated that the differences in the microbial succession across the pits of different ages might be related to the microbial composition of the pit mud. In addition, species that could not be identified in the existing database were also detected in ZP100S, indicating that a unique microbial community structure formed during the long-term fermentation process (Li et al., 2022). Thus, obtaining new microbial resources from the complex brewing environment and utilizing them can benefit the quality improvement of Baijiu.

A total of seven genera, namely *Lactobacillus*, *Weissella*, *Aspergillus*, *Trichosporon*, *Kazachstania*, *Issatchenkia*, and *Pichia,* were detected in the three kinds of ZPs, and all played important roles during the fermentation of strong-flavor Baijiu. *Lactobacillus* and *Weissella* can metabolize substrates to produce organic acids, such as lactic acid and acetic acid, which are precursors for the synthesis of flavor components (Wang S. H. et al., 2023). *Aspergillus* has the ability to produce flavor compounds owing to its various enzyme metabolic capabilities (Yang et al., 2020). *Trichosporon* can catalyze the formation of esters with the assistance of a highly active lipase (Tang et al., 2019). The fermentation of *Kazachstania* can accelerate the accumulation of volatile flavor compounds, including esters, acids, and alcohols (Illse et al., 2017). *Issatchenkia*, the dominant functional fungus in ZPs, can produce various esters and other aromatic compounds during fermentation. Meanwhile, it possesses significant ethanol production capability (Guan et al., 2023). *Pichia* can translate sucrose and glucose to various aromatic substances, such as ethyl acetate, enhancing the flavor of Baijiu (Zhang et al., 2017).

Physicochemical properties are not only important indicators for evaluating the fermentation status of Baijiu but also potential driving factors for microbial community succession during fermentation (Xu et al., 2023). The results showed that the acidity content increased the most in ZP35S, which resulted from the metabolism of acid-producing microorganisms, such as *Lactobacillus* and *Acetobacter* (Li et al., 2016). The change in acidity was related to the abundance and metabolic strength of the acid-producing microorganisms. *Lactobacillus* was the dominant bacterial genus, and its relative abundance was higher in ZP35S than in ZP5S and ZP100S. In addition, the RDA showed that acidity was positively correlated with *Lactobacillus*, indicating that *Lactobacillus* was the main bacteria affecting the acidity of the ZPs during the fermentation of strong-flavor Baijiu. This finding is consistent with that of reported studies (He et al., 2022). In addition, higher acidity could inhibit the growth of other microorganisms and reduce microbial diversity, changing the composition of the microbial community during the fermentation of ZPs (Luo et al., 2023). On the 7th day of the fermentation, the moisture content in the ZPs sharply increased, while the starch content notably decreased, probably because of sufficient oxygen present during the early stage of the fermentation. This allowed the rapid growth and propagation of aerobic microorganisms such as Issatchenkia, Kazachstania, Pichia, Saccharomyces, Scedosporium, and Byssochlamys, which consumed starch and produced water (Luo et al., 2023). Water is an integral part of biochemical reactions and acts as an effective solvent for microorganism growth and metabolite production (Liu et al., 2021). The RDA showed that six genera were positively correlated with starch, namely *Thermoactinomyces*, *Staphylococcus*, *Kroppenstedtia*, *Bacillus*, *Saccharomycopsis,* and *Thermomyces*, indicating that these microorganisms may have the ability to produce amylase (Hu et al., 2021). On the 7th day, the reducing sugar content in ZP5S increased, likely due to the extensive hydrolysis of starch by amylase secreted from ester-producing microorganisms. The production rate of reducing sugars was much faster than its consumption rate (McFeeters, 2004). The reducing sugar content in the ZPs from the different pits tended to stabilize in the later stage of the fermentation, which is similar to the results of Guan and Lin (Guan et al., 2020; Lin et al., 2025), showing a dynamic balance between production and consumption (Hao et al., 2021). In conclusion, the dynamic changes in the physicochemical properties of the pits of different ages could reflect microbial metabolism during Baijiu fermentation and provide a basis for the scientific control of the fermentation process of strong-flavor Baijiu (Liu et al., 2021). Owing to the environmental changes, such as high moisture and high acidity, the unsuitable microorganisms, including *Bacillus*, *Acetobacter,* and *Kroppenstedtia,* were gradually eliminated, and the microbial diversity decreased. On the contrary, *Lactobacillus*, *Aspergillus,* and *Issatchenkia* gradually increased.

Strong-flavor Baijiu fermentation is a complex process in which numerous microorganisms work together. During fermentation, a variety of enzymes produced by these microorganisms catalyze substrates to form a wide range of flavor compounds (Wang et al., 2020). It is the diversity of microbial communities that drives the abundance of volatile flavor components (He et al., 2020). A total of 177 volatile flavor components were detected in all samples, with the top three being esters, alcohols, and acids. This finding is consistent with that of previous research (Hu et al., 2021). Esters mainly provide fruity and floral flavors, giving Baijiu its unique flavor characteristics (Xu Y. Q. et al., 2022). Alcohols typically provide fruity and sweet flavors (Wang J. S. et al., 2022). Acids can reduce the bitterness and increase the sweetness of Baijiu (Wu Y. S. et al., 2022). In summary, esters, alcohols, and acids play important roles in the flavor and quality of strong-flavor Baijiu. Ethyl hexanoate, ethyl acetate, ethyl butyrate, and ethyl lactate are the four most important esters in strong-flavor Baijiu (Guan et al., 2023). Among the dominant genera in the three kinds of ZPs, 15 genera, such as *Lactobacillus*, *Caproiiciproducers*, *Clostridium_senu_stricto_1*, *Pichia*, *Issatchenkia*, *Saccharomyces,* and *Aspergillus,* were significantly positively correlated with the four major ethyl esters (*p* < 0.05 or 0.01). This correlation was mainly because these microorganisms can directly or indirectly synthesize the four major ethyl esters or their precursors. *Aspergillus*, an important filamentous fungus, can produce enzymes and degrade starch into fermentable sugars, which is conducive to the fermentation of strong-flavor Baijiu (Chen et al., 2014). *Bacillus* also can convert starch into reducing sugars, providing a carbon source for other microorganisms (Zou et al., 2018). *Lactobacillus* and *Weissella* consume sugars to produce lactic acid and acetic acid, which further react with ethanol to form ethyl lactate and ethyl acetate, respectively (Tian et al., 2020; Wang S. H. et al., 2023). *Caproiciproducens* can produce hexanoic acid using ethanol as the substrate (Wang et al., 2021). *Clostridium* can produce acetic acid or butyric acid (Wu L. T. et al., 2022). In addition, some volatile flavor components were positively correlated with at least one dominant genus, likely due to the formation of substances requiring the joint participation of several microorganisms (Hu et al., 2020).

## 5 Conclusion

In conclusion, this study systematically revealed the dynamic changes in the physicochemical properties, volatile flavor components, and microbial communities of ZPs during the fermentation of strong-flavor Baijiu with different pit ages. During the fermentation of the ZPs, the acidity and moisture content increased, while the pH, starch, and reducing sugar content decreased. In the 100-year-old pit, the ethyl acetate content was the lowest, while the ethyl caproate content was relatively higher. A total of 690 bacterial genera and 155 fungal genera were detected. The diversity of the bacteria was higher than that of the fungi, and the microbial community composition of ZP100S was the most abundant among the three kinds of ZPs. Firmicutes and Ascomycota were the most dominant bacterial and fungal phyla, and *Lactobacillus* was the dominant bacterial genus. *Lactobacillus*, *Capriciproducers*, *Pichia*, *Issatchenkia*, *Saccharomyces*, and *Aspellus* showed a significant positive correlation with the four important esters. In addition, some volatile flavor components were correlated with at least one dominant genus. This study is an important contribution to research on microbial diversity in strong-flavor Baijiu. Furthermore, the findings provide a theoretical basis for technological control to improve the quality of strong-flavor Baijiu.

## Data Availability

The original contributions presented in the study are publicly available. This data can be found here: [https://www.ncbi.nlm.nih.gov/bioproject/PRJNA1208917].

## Footnotes

^1^ https://www.majorbio.com
