# Supplementary material for: Effects of pits of different ages on ethyl acetate and its metabolism-related microorganisms during strong-flavor Baijiu fermentation
Source: Front Microbiol. 2025 Jan 27;16:1532869. doi: 10.3389/fmicb.2025.1532869 (PMC11807979; doi:10.3389/fmicb.2025.1532869)
Supplement: Supplementary file 1 [file Data_Sheet_1.docx]

**Effects of pits of different ages on ethyl acetate and its metabolism-related microorganisms during strong-flavor Baijiu fermentation**

**Fig. S1**. Microbial community structure of ZPs during the fermentation of Baijiu (phylum level). (A) Bacteria. (B) Fungi.

**Fig. S2**. RDA or CA of dominant bacteria genera and physicochemical properties. (A) ZP5S. (B) ZP35S. (C) ZP100S.

**Table S1**. Volatile flavor compounds detected in ZPs.

| Number | Compound | Category |
| --- | --- | --- |
| 1 | 1,1'-Oxybisethanol diacetate | Esters |
| 2 | Hexamethylene diacrylate | Esters |
| 3 | 2-Methylpentanoic anhydride | Esters |
| 4 | 2,2,4-Trimethyl-1,3-pentanediol diisobutyrate | Esters |
| 5 | Methyl 2,4-dimethyl-3-oxopentanoate | Esters |
| 6 | 3-Hydroxy-2,2,4-trimethylpentyl isobutyrate | Esters |
| 7 | Ethyl 2-hydroxy-3-phenylpropanoate | Esters |
| 8 | Ethyl 2-hydroxy-3-methylbutanoate | Esters |
| 9 | Furfuryl pentanoate | Esters |
| 10 | 3-(Methylthio)propyl acetate | Esters |
| 11 | Ethyl 3-phenylpropionate | Esters |
| 12 | 2-Butanol-3-methyl acetate | Esters |
| 13 | Ethyl 3-methylthiopropionate | Esters |
| 14 | Ethyl 3-hydroxyoctanoate | Esters |
| 15 | 3-Ethoxypropyl acetate | Esters |
| 16 | (E)-Ethyl 3-ethoxy-2-propenoate | Esters |
| 17 | Ethyl phenylbutyrate | Esters |
| 18 | Methyl 4-methyl valerate | Esters |
| 19 | Ethyl 8-nonenoate | Esters |
| 20 | Ethyl dl-2-hydroxycaproate | Esters |
| 21 | Ethyl 2-hydroxy-4-methylvalerate | Esters |
| 22 | Ethyl benzoate | Esters |
| 23 | Ethyl phenylacetate | Esters |
| 24 | 1-Methoxy-2-propyl acetate | Esters |
| 25 | Ethyl propionate | Esters |
| 26 | Ethyl hydrogen succinate | Esters |
| 27 | Diethyl succinate | Esters |
| 28 | beta-Phenylethyl butyrate | Esters |
| 29 | Butyl butyrate | Esters |
| 30 | Ethyl butyrate | Esters |
| 31 | Isoamyl butyrate | Esters |
| 32 | Butyl butyryllactate | Esters |
| 33 | Diethyl pimelate | Esters |
| 34 | Ethyl enanthate | Esters |
| 35 | Benzylcarbinyl caproate | Esters |
| 36 | Propyl caproate | Esters |
| 37 | Hexyl hexanoate | Esters |
| 38 | Methyl hexoate | Esters |
| 39 | Furfuryl hexanoate | Esters |
| 40 | Ethyl hexanoate | Esters |
| 41 | Isobutyl hexanoate | Esters |
| 42 | 3-Methylbutyl hexanoate | Esters |
| 43 | Heptyl formate | Esters |
| 44 | 2-Phenylethyl methoxyacetate | Esters |
| 45 | Methyl methoxyacetate | Esters |
| 46 | Diethyl azelate | Esters |
| 47 | Ethyl nonanoate | Esters |
| 48 | Phenylethyl lactate | Esters |
| 49 | Propyl lactate | Esters |
| 50 | Butyl lactate | Esters |
| 51 | Hexyl lactate | Esters |
| 52 | Ethyl lactate | Esters |
| 53 | R-(+)-isobutyl lactate | Esters |
| 54 | Isoamyl lactate | Esters |
| 55 | Ethyl docosanoate | Esters |
| 56 | Ethyl 3-hydroxytridecanoate | Esters |
| 57 | Pentyl 2-hydroxypropanoate | Esters |
| 58 | Ethyl hydrogen glutarate | Esters |
| 59 | Diethyl glutarate | Esters |
| 60 | Propyl valerate | Esters |
| 61 | Furfuryl pentanoate | Esters |
| 62 | Ethyl valerate | Esters |
| 63 | Diethyl suberate | Esters |
| 64 | Ethyl caprylate | Esters |
| 65 | Dihydro-5-pentyl-2(3H)-furanone | Esters |
| 66 | Ethyl 2-hydroxy-3-phenylpropanoate | Esters |
| 67 | 3-(Methylthio)propyl acetate | Esters |
| 68 | 2-Phenylethyl acetate | Esters |
| 69 | Propyl acetate | Esters |
| 70 | Ethyl butanoate | Esters |
| 71 | Heptyl acetate | Esters |
| 72 | Hexyl acetate | Esters |
| 73 | Pentyl acetate | Esters |
| 74 | Ethyl acetate | Esters |
| 75 | Isoamyl acetate | Esters |
| 76 | 2-Phenylethyl isobutyrate | Esters |
| 77 | Ethyl 4-methylvalerate | Esters |
| 78 | Isoamyl isovalerate | Esters |
| 79 | Isopentyl valerate | Esters |
| 80 | Ethyl palmitate | Esters |
| 81 | [R-(R*, R*)]-2,3-Butanediol | Alcohols |
| 82 | 3-Ethoxy-1-propanol | Alcohols |
| 83 | 1,7-Octadien-3-ol | Alcohols |
| 84 | 1-Eicosanol | Alcohols |
| 85 | 1-Hexanol | Alcohols |
| 86 | 1-Nonanol | Alcohols |
| 87 | 1-Pentanol | Alcohols |
| 88 | 2,5-Dimethyl-2,5-hexanediol | Alcohols |
| 89 | 2,6-Dimethyl-4-heptanol | Alcohols |
| 90 | 2-Butanol | Alcohols |
| 91 | (E)-2-Decen-1-ol | Alcohols |
| 92 | 2-Hexanol | Alcohols |
| 93 | 2-Nonanol | Alcohols |
| 94 | 2-Nonen-1-ol | Alcohols |
| 95 | 2-Ethyl-1-hexanol | Alcohols |
| 96 | 3-Methyl-1-butanol | Alcohols |
| 97 | 3-Octanol | Alcohols |
| 98 | 2,7-Dimethyl-4,5-octanediol | Alcohols |
| 99 | 4-Phenyl-3-buten-2-ol | Alcohols |
| 100 | 6-Undecanol | Alcohols |
| 101 | beta-Ethylphenethyl alcohol | Alcohols |
| 102 | 2-Pentanol | Alcohols |
| 103 | 1-Methyl-4-(1-methylethyl)-cyclohexanol | Alcohols |
| 104 | dl-Menthol | Alcohols |
| 105 | Benzyl alcohol | Alcohols |
| 106 | Phenylethyl alcohol | Alcohols |
| 107 | Propylene glycol | Alcohols |
| 108 | 2,6-Dimethyl-4-heptanol | Alcohols |
| 109 | (E)-2-Hepten-1-ol | Alcohols |
| 110 | (E)-2-Nonen-1-ol | Alcohols |
| 111 | Trans-2-U0ecen-1-ol | Alcohols |
| 112 | (E)-2-Octen-1-ol | Alcohols |
| 113 | 2-Furanmethanol | Alcohols |
| 114 | Ledol | Alcohols |
| 115 | 1-Octen-3-ol | Alcohols |
| 116 | Tris(1-methylethyl)silanol | Alcohols |
| 117 | 1-Octanol | Alcohols |
| 118 | Ethanol | Alcohols |
| 119 | 2-Methyl-1-propanol | Alcohols |
| 120 | 2-Ethyl-1-hexanol | Alcohols |
| 121 | 1-Heptanol | Alcohols |
| 122 | 1-Decanol | Alcohols |
| 123 | 2,4-Dimethylpentanoic acid | Acids |
| 124 | 2-Methylhexanoic acid | Acids |
| 125 | 2-Methylpentanoic acid | Acids |
| 126 | 2-Ethyl-2-hydroxybutyric acid | Acids |
| 127 | 4-Methylpentanoic acid | Acids |
| 128 | 5-Methylhexanoic acid | Acids |
| 129 | 6-Methylheptanoic acid | Acids |
| 130 | Propanoic acid | Acids |
| 131 | Acetic acid | Acids |
| 132 | Butanoic acid | Acids |
| 133 | Heptanoic acid | Acids |
| 134 | Heptanoic anhydride | Acids |
| 135 | Hexanoic acid | Acids |
| 136 | Hexanoic anhydride | Acids |
| 137 | Nonanoic acid | Acids |
| 138 | Undecanoic acid | Acids |
| 139 | Pentanoic acid | Acids |
| 140 | Octanoic acid | Acids |
| 141 | 3-Methylbutanoic acid | Acids |
| 142 | 2-Ethylhexanoic acid | Acids |
| 143 | n-Decanoic acid | Acids |
| 144 | 2-Heptanone | Ketones |
| 145 | 2-Nonanone | Ketones |
| 146 | 2-Octanone | Ketones |
| 147 | Acetoin | Ketones |
| 148 | 3-Octanone | Ketones |
| 149 | 5-Hydroxy-2-methyl-3-hexanone | Ketones |
| 150 | Acetone | Ketones |
| 151 | Dicyclohexylmethanone | Ketones |
| 152 | 6-Methylhept-5-en-2-one | Ketones |
| 153 | 6,10-dimethyl-, (E)-5,9-Undecadien-2-one | Ketones |
| 154 | 2,4,5-Trimethylbenzaldehyde | Aldehydes |
| 155 | 2,4,6-Trimethylbenzaldehyde | Aldehydes |
| 156 | 3,4-Dimethylbenzaldehyde | Aldehydes |
| 157 | 5-Methyl-2-furancarboxaldehyde | Aldehydes |
| 158 | Benzaldehyde | Aldehydes |
| 159 | Benzeneacetaldehyde | Aldehydes |
| 160 | 4-Methylbenzaldehyde | Aldehydes |
| 161 | Decanal | Aldehydes |
| 162 | Furfural | Aldehydes |
| 163 | Dodecanal | Aldehydes |
| 164 | Nonanal | Aldehydes |
| 165 | 2,4-Di-tert-butylphenol | Phenols |
| 166 | 2-Methoxy-4-propylphenol | Phenols |
| 167 | Creosol | Phenols |
| 168 | 2-Methoxy-5-methylphenol | Phenols |
| 169 | 4-Ethylphenol | Phenols |
| 170 | 4-Ethyl-2-methoxyphenol | Phenols |
| 171 | 2-Methoxy-4-vinylphenol | Phenols |
| 172 | Phenol | Phenols |
| 173 | 2-(1,1-dimethylethyl)-4-methylphenol | Phenols |
| 174 | p-Cresol | Phenols |
| 175 | 1,2-dimethoxybenzene | Others |
| 176 | 2-Methyloctane | Others |
| 177 | Tetradecane | Others |

**Table S2**. The contents of volatile flavor components during the fermentation ZPs.

| Samples | Content (μg/g) | | | | | | |
| --- | --- | --- | --- | --- | --- | --- | --- |
|  | Esters | Alcohols | Acids | Ketones | Aldehydes | Phenols | Others |
| ZP5S0 | 16.55 ± 0.26 | 2.98 ± 0.07 | 37.97 ± 0.75 | 3.56 ± 0.09 | 0.75 ± 0.04 | 9.4 ± 0.65 | ND |
| ZP5S7 | 41.3 ± 0.22 | 10.32 ± 1.07 | 28.22 ± 0.53 | 3.64 ± 0.09 | 0.81 ± 0.06 | 6.65 ± 0.2 | 0.02 ± 0 |
| ZP5S14 | 41.53 ± 0.64 | 12.42 ± 0.23 | 25.83 ± 0.26 | 3.63 ± 0.03 | 0.63 ± 0.01 | 6.93 ± 0.78 | ND |
| ZP5S21 | 59.44 ± 6.51 | 14.97 ± 2.07 | 30.91 ± 3.19 | 3.45 ± 0.09 | 0.59 ± 0.02 | 8.17 ± 0.38 | 0.01 ± 0 |
| ZP5S30 | 53.79 ± 1.41 | 16.21 ± 0.62 | 33.02 ± 0.31 | 3.56 ± 0.05 | 0.83 ± 0.05 | 8.03 ± 0.39 | 0.01 ± 0 |
| ZP5S40 | 54.19 ± 0.84 | 18.31 ± 1.35 | 38.19 ± 1.04 | 3.26 ± 0.02 | 0.76 ± 0.03 | 9.63 ± 1.32 | 0.01 ± 0 |
| ZP5S50 | 50.26 ± 0.55 | 16.41 ± 0.09 | 32.24 ± 0.16 | 3.73 ± 0.08 | 0.84 ± 0.06 | 10.89 ± 0.47 | ND |
| ZP5S60 | 57.44 ± 1.94 | 16.79 ± 0.45 | 34.86 ± 0.69 | 3.39 ± 0.06 | 0.76 ± 0.1 | 11.58 ± 0.72 | ND |
| ZP35S0 | 6.94 ± 0.43 | 2.2 ± 0.08 | 6.13 ± 0.37 | 4.31 ± 0.04 | 1.07 ± 0.05 | 4.74 ± 0.41 | ND |
| ZP35S7 | 18.59 ± 0.61 | 12.44 ± 0.65 | 5.99 ± 0.19 | 4.12 ± 0.08 | 0.29 ± 0.04 | 5.99 ± 0.16 | ND |
| ZP35S14 | 21.01 ± 0.61 | 13.02 ± 1.35 | 5.32 ± 0.1 | 3.98 ± 0.02 | 0.47 ± 0.05 | 7.15 ± 1.05 | ND |
| ZP35S21 | 28.69 ± 7.24 | 18.04 ± 5.1 | 7.63 ± 2.17 | 3.17 ± 0.37 | 1.16 ± 0.27 | 8.6 ± 1.92 | ND |
| ZP35S30 | 21.82 ± 1.68 | 14.47 ± 1.94 | 5.45 ± 0.48 | 4.08 ± 0.06 | 0.53 ± 0.07 | 5.01 ± 0.36 | ND |
| ZP35S40 | 23.51 ± 1.64 | 12.84 ± 1.82 | 4.83 ± 0.17 | 3.93 ± 0.13 | 1.1 ± 0.07 | 5.87 ± 0.57 | ND |
| ZP35S50 | 26.96 ± 1.45 | 13.06 ± 1.15 | 5.9 ± 0.36 | 3.84 ± 0.16 | 0.77 ± 0.01 | 5.77 ± 0.41 | ND |
| ZP35S60 | 27.98 ± 1.65 | 13.03 ± 0.74 | 5.8 ± 0.36 | 3.8 ± 0.14 | 0.57 ± 0.06 | 6.82 ± 1.34 | ND |
| ZP100S0 | 8.41 ± 0.23 | 2.65 ± 0.07 | 15.79 ± 0.98 | 2.78 ± 0.06 | 1.13 ± 0.06 | 4.12 ± 0.92 | ND |
| ZP100S7 | 38.38 ± 1.25 | 9.55 ± 0.65 | 24.64 ± 1.37 | 3.16 ± 0.11 | 1.05 ± 0.06 | 6.67 ± 0.41 | ND |
| ZP100S14 | 41.39 ± 1.86 | 12.56 ± 0.91 | 33.9 ± 0.94 | 3.15 ± 0.08 | 0.88 ± 0.02 | 9.25 ± 0.94 | 0.01 ± 0 |
| ZP100S21 | 52.61 ± 1.98 | 8.63 ± 0.19 | 47.2 ± 2.11 | 3.28 ± 0.07 | 0.94 ± 0.04 | 6.75 ± 0.25 | ND |
| ZP100S30 | 42.59 ± 1.89 | 17.93 ± 1.3 | 60.28 ± 4.73 | 2.01 ± 0.1 | 0.97 ± 0.07 | 6.95 ± 0.71 | 0.01 ± 0 |
| ZP100S40 | 44.01 ± 1.11 | 11.26 ± 0.34 | 61.79 ± 0.71 | 3.18 ± 0.09 | 1.01 ± 0.13 | 8.26 ± 1.06 | ND |
| ZP100S50 | 54.2 ± 2.66 | 17.76 ± 0.44 | 68.35 ± 3.32 | 3.04 ± 0.14 | 0.84 ± 0.05 | 10.1 ± 2.58 | ND |
| ZP100S60 | 68.96 ± 1.43 | 16.35 ± 0.74 | 62.24 ± 4.47 | 3.28 ± 0.29 | 0.83 ± 0.12 | 8.03 ± 1.59 | ND |

Note: ND represented not detected. Data are presented as means ± standard deviations (n = 3).

**Table S3**. Bacterial diversity analysis.

| Samples | Sobs | Shannon | Simpson | Ace | Chao | Coverage |
| --- | --- | --- | --- | --- | --- | --- |
| ZP5S0 | 100 | 2.56 | 0.13 | 124.69 | 125.09 | 99.92% |
| ZP5S7 | 184 | 2.14 | 0.27 | 224.96 | 222.33 | 99.84% |
| ZP5S14 | 30 | 0.95 | 0.45 | 35.05 | 33.00 | 99.98% |
| ZP5S21 | 43 | 1.15 | 0.40 | 282.31 | 100.75 | 99.93% |
| ZP5S30 | 32 | 1.60 | 0.27 | 72.40 | 44.00 | 99.97% |
| ZP5S40 | 40 | 1.23 | 0.42 | 99.57 | 70.00 | 99.95% |
| ZP5S50 | 83 | 1.09 | 0.47 | 126.45 | 139.10 | 99.88% |
| ZP5S60 | 120 | 1.35 | 0.39 | 204.46 | 156.12 | 99.85% |
| ZP35S0 | 91 | 1.65 | 0.35 | 107.46 | 110.00 | 99.94% |
| ZP35S7 | 153 | 1.31 | 0.44 | 202.52 | 206.45 | 99.83% |
| ZP35S14 | 22 | 0.11 | 0.97 | 30.06 | 31.33 | 99.97% |
| ZP35S21 | 24 | 0.20 | 0.93 | 54.57 | 39.00 | 99.97% |
| ZP35S30 | 35 | 0.49 | 0.76 | 92.47 | 55.00 | 99.95% |
| ZP35S40 | 50 | 0.61 | 0.70 | 66.61 | 65.55 | 99.94% |
| ZP35S50 | 38 | 0.47 | 0.76 | 94.89 | 60.67 | 99.94% |
| ZP35S60 | 28 | 0.39 | 0.81 | 45.82 | 35.50 | 99.97% |
| ZP100S0 | 314 | 2.75 | 0.17 | 339.55 | 331.07 | 99.85% |
| ZP100S7 | 836 | 3.88 | 0.09 | 1038.60 | 1051.86 | 99.22% |
| ZP100S14 | 548 | 1.49 | 0.61 | 766.19 | 749.03 | 99.32% |
| ZP100S21 | 894 | 4.02 | 0.08 | 1091.13 | 1101.72 | 99.21% |
| ZP100S30 | 793 | 2.96 | 0.22 | 983.60 | 961.11 | 99.26% |
| ZP100S40 | 831 | 3.66 | 0.11 | 1041.27 | 1018.08 | 99.22% |
| ZP100S50 | 811 | 3.54 | 0.11 | 991.54 | 986.52 | 99.26% |
| ZP100S60 | 894 | 4.34 | 0.05 | 1063.36 | 1028.63 | 99.29% |

**Table S4**. Fungal diversity analysis.

| Samples | sobs | shannon | simpson | ace | chao | coverage |
| --- | --- | --- | --- | --- | --- | --- |
| ZP5S0 | 66 | 1.20 | 0.40 | 71.64 | 71.00 | 99.97% |
| ZP5S7 | 55 | 1.24 | 0.39 | 59.45 | 56.75 | 99.98% |
| ZP5S14 | 39 | 0.95 | 0.59 | 59.72 | 48.17 | 99.97% |
| ZP5S21 | 59 | 1.10 | 0.42 | 123.13 | 95.14 | 99.94% |
| ZP5S30 | 44 | 0.51 | 0.80 | 62.20 | 61.50 | 99.96% |
| ZP5S40 | 78 | 1.50 | 0.41 | 83.16 | 82.20 | 99.98% |
| ZP5S50 | 62 | 1.04 | 0.53 | 71.10 | 70.25 | 99.97% |
| ZP5S60 | 39 | 1.63 | 0.43 | 42.33 | 40.50 | 99.99% |
| ZP35S0 | 81 | 1.46 | 0.40 | 89.90 | 94.00 | 99.97% |
| ZP35S7 | 89 | 1.84 | 0.23 | 95.89 | 96.58 | 99.96% |
| ZP35S14 | 64 | 1.56 | 0.29 | 73.41 | 73.00 | 99.97% |
| ZP35S21 | 66 | 1.55 | 0.32 | 73.04 | 75.33 | 99.98% |
| ZP100Z30 | 45 | 1.34 | 0.44 | 46.93 | 46.00 | 99.99% |
| ZP35S40 | 47 | 1.41 | 0.45 | 50.31 | 50.00 | 99.99% |
| ZP35S50 | 40 | 1.22 | 0.60 | 66.26 | 50.00 | 99.99% |
| ZP35S60 | 50 | 1.69 | 0.42 | 52.13 | 53.00 | 99.99% |
| ZP100S0 | 69 | 1.09 | 0.56 | 82.86 | 77.75 | 99.96% |
| ZP100S7 | 112 | 2.19 | 0.20 | 186.80 | 152.62 | 99.92% |
| ZP100S14 | 100 | 2.37 | 0.17 | 112.95 | 115.11 | 99.96% |
| ZP100S21 | 120 | 2.89 | 0.09 | 131.59 | 131.33 | 99.96% |
| ZP100S30 | 128 | 2.61 | 0.12 | 152.39 | 153.00 | 99.93% |
| ZP100S40 | 152 | 2.91 | 0.09 | 163.01 | 162.06 | 99.95% |
| ZP100S50 | 110 | 2.63 | 0.12 | 116.68 | 117.86 | 99.97% |
| ZP100S60 | 108 | 2.27 | 0.19 | 132.78 | 126.75 | 99.94% |

**Table S5**. The correlation coefficient between dominant genera and volatile flavor compounds of ZP5S.

| Number of volatile flavor compounds | Volatile flavor compounds | Number  of genera | Correlation coefficient |
| --- | --- | --- | --- |
| 1 | 1,1'-Oxybisethanol diacetate | B1 | -0.736 |
| 1 | 1,1'-Oxybisethanol diacetate | F2 | 0.913 |
| 1 | 1,1'-Oxybisethanol diacetate | F5 | -0.862 |
| 2 | Hexamethylene diacrylate | B1 | -0.762 |
| 2 | Hexamethylene diacrylate | B13 | -0.733 |
| 2 | Hexamethylene diacrylate | B2 | -0.833 |
| 2 | Hexamethylene diacrylate | B9 | 0.952 |
| 2 | Hexamethylene diacrylate | F17 | -0.731 |
| 2 | Hexamethylene diacrylate | F6 | -0.85 |
| 6 | 3-Hydroxy-2,2,4-trimethylpentyl isobutyrate | F17 | -0.778 |
| 7 | Ethyl 2-hydroxy-3-phenylpropanoate | B10 | 0.774 |
| 7 | Ethyl 2-hydroxy-3-phenylpropanoate | F2 | -0.812 |
| 7 | Ethyl 2-hydroxy-3-phenylpropanoate | F5 | 0.71 |
| 11 | Ethyl 3-phenylpropionate | B13 | -0.764 |
| 11 | Ethyl 3-phenylpropionate | B2 | -0.81 |
| 11 | Ethyl 3-phenylpropionate | B9 | 0.857 |
| 12 | 2-Butanol-3-methyl acetate | B1 | 0.764 |
| 12 | 2-Butanol-3-methyl acetate | F2 | -0.791 |
| 13 | Ethyl 3-methylthiopropionate | B1 | -0.846 |
| 13 | Ethyl 3-methylthiopropionate | B9 | 0.846 |
| 13 | Ethyl 3-methylthiopropionate | F2 | 0.873 |
| 13 | Ethyl 3-methylthiopropionate | F5 | -0.791 |
| 13 | Ethyl 3-methylthiopropionate | F6 | -0.7 |
| 14 | Ethyl 3-hydroxyoctanoate | B8 | -0.753 |
| 17 | Ethyl phenylbutyrate | B10 | 0.707 |
| 17 | Ethyl phenylbutyrate | B14 | -0.826 |
| 17 | Ethyl phenylbutyrate | B7 | -0.766 |
| 17 | Ethyl phenylbutyrate | F10 | 0.826 |
| 17 | Ethyl phenylbutyrate | F23 | 0.729 |
| 20 | Ethyl dl-2-hydroxycaproate | B13 | -0.764 |
| 20 | Ethyl dl-2-hydroxycaproate | B2 | -0.762 |
| 20 | Ethyl dl-2-hydroxycaproate | B9 | 0.952 |
| 20 | Ethyl dl-2-hydroxycaproate | F17 | -0.79 |
| 20 | Ethyl dl-2-hydroxycaproate | F6 | -0.755 |
| 22 | Ethyl benzoate | B1 | -0.738 |
| 22 | Ethyl benzoate | B13 | -0.764 |
| 22 | Ethyl benzoate | B2 | -0.833 |
| 22 | Ethyl benzoate | B9 | 0.929 |
| 23 | Ethyl phenylacetate | B1 | -0.738 |
| 23 | Ethyl phenylacetate | B13 | -0.764 |
| 23 | Ethyl phenylacetate | B2 | -0.833 |
| 23 | Ethyl phenylacetate | B9 | 0.929 |
| 26 | Ethyl hydrogen succinate | F5 | -0.786 |
| 26 | Ethyl hydrogen succinate | F6 | -0.755 |
| 27 | Diethyl succinate | B9 | 0.81 |
| 27 | Diethyl succinate | F17 | -0.838 |
| 28 | beta-Phenylethyl butyrate | F17 | -0.711 |
| 28 | beta-Phenylethyl butyrate | F6 | -0.735 |
| 29 | Butyl butyrate | B13 | 0.756 |
| 33 | Diethyl pimelate | B10 | 0.733 |
| 34 | Ethyl enanthate | B10 | 0.857 |
| 36 | Propyl caproate | B13 | -0.764 |
| 36 | Propyl caproate | B9 | 0.738 |
| 36 | Propyl caproate | F17 | -0.755 |
| 42 | 3-Methylbutyl hexanoate | B8 | -0.753 |
| 43 | Heptyl formate | F23 | 0.702 |
| 45 | Methyl methoxyacetate | B10 | -0.952 |
| 45 | Methyl methoxyacetate | F5 | -0.714 |
| 46 | Diethyl azelate | B13 | -0.733 |
| 46 | Diethyl azelate | B9 | 0.762 |
| 46 | Diethyl azelate | F14 | -0.857 |
| 46 | Diethyl azelate | F17 | -0.874 |
| 46 | Diethyl azelate | F6 | -0.719 |
| 48 | Phenylethyl lactate | B2 | -0.764 |
| 48 | Phenylethyl lactate | F23 | 0.768 |
| 49 | Propyl lactate | B2 | -0.886 |
| 49 | Propyl lactate | B9 | 0.707 |
| 52 | Ethyl lactate | B10 | -0.905 |
| 52 | Ethyl lactate | F5 | -0.738 |
| 53 | R-(+)-isobutyl lactate | F17 | 0.817 |
| 54 | Isoamyl lactate | B13 | -0.764 |
| 54 | Isoamyl lactate | B9 | 0.786 |
| 54 | Isoamyl lactate | F14 | -0.81 |
| 54 | Isoamyl lactate | F17 | -0.898 |
| 59 | Diethyl glutarate | B8 | -0.702 |
| 64 | Ethyl caprylate | B10 | 0.786 |
| 65 | Dihydro-5-pentyl-2(3H)-furanone | B2 | -0.857 |
| 65 | Dihydro-5-pentyl-2(3H)-furanone | B9 | 0.762 |
| 65 | Dihydro-5-pentyl-2(3H)-furanone | F2 | 0.738 |
| 65 | Dihydro-5-pentyl-2(3H)-furanone | F5 | -0.786 |
| 65 | Dihydro-5-pentyl-2(3H)-furanone | F6 | -0.755 |
| 66 | Ethyl 2-hydroxy-3-phenylpropanoate | B1 | -0.786 |
| 66 | Ethyl 2-hydroxy-3-phenylpropanoate | F2 | 0.939 |
| 66 | Ethyl 2-hydroxy-3-phenylpropanoate | F5 | -0.913 |
| 66 | Ethyl 2-hydroxy-3-phenylpropanoate | F6 | -0.753 |
| 67 | 3-(Methylthio)propyl acetate | B8 | -0.753 |
| 68 | 2-Phenylethyl acetate | B13 | -0.764 |
| 68 | 2-Phenylethyl acetate | B2 | -0.881 |
| 68 | 2-Phenylethyl acetate | B9 | 0.833 |
| 72 | Hexyl acetate | B13 | -0.733 |
| 72 | Hexyl acetate | B2 | -0.833 |
| 72 | Hexyl acetate | B9 | 0.833 |
| 74 | Ethyl acetate | B13 | -0.764 |
| 74 | Ethyl acetate | F10 | 0.714 |
| 74 | Ethyl acetate | F14 | -0.714 |
| 75 | Isoamyl acetate | B13 | -0.733 |
| 75 | Isoamyl acetate | B2 | -0.833 |
| 75 | Isoamyl acetate | B9 | 0.714 |
| 77 | Ethyl 4-methylvalerate | F10 | 0.733 |
| 77 | Ethyl 4-methylvalerate | F14 | -0.733 |
| 79 | Isopentyl valerate | F10 | 0.764 |
| 79 | Isopentyl valerate | F14 | -0.846 |
| 80 | Ethyl palmitate | B1 | -0.838 |
| 80 | Ethyl palmitate | B13 | -0.753 |
| 80 | Ethyl palmitate | B2 | -0.874 |
| 80 | Ethyl palmitate | B9 | 0.994 |
| 80 | Ethyl palmitate | F6 | -0.759 |
| 81 | [R-(R*, R*)]-2,3-Butanediol | B10 | 0.774 |
| 81 | [R-(R*, R*)]-2,3-Butanediol | F2 | -0.812 |
| 81 | [R-(R*, R*)]-2,3-Butanediol | F5 | 0.71 |
| 82 | 3-Ethoxy-1-propanol | B2 | -0.764 |
| 82 | 3-Ethoxy-1-propanol | F10 | 0.873 |
| 82 | 3-Ethoxy-1-propanol | F23 | 0.768 |
| 85 | 1-Hexanol | B1 | -0.81 |
| 85 | 1-Hexanol | B13 | -0.764 |
| 85 | 1-Hexanol | B2 | -0.857 |
| 85 | 1-Hexanol | B9 | 1 |
| 85 | 1-Hexanol | F6 | -0.707 |
| 86 | 1-Nonanol | B13 | -0.764 |
| 86 | 1-Nonanol | B2 | -0.833 |
| 86 | 1-Nonanol | B9 | 0.738 |
| 86 | 1-Nonanol | F17 | -0.755 |
| 89 | 2,6-Dimethyl-4-heptanol | B1 | 0.709 |
| 93 | 2-Nonanol | F10 | 0.881 |
| 93 | 2-Nonanol | F23 | 0.778 |
| 95 | 2-Ethyl-1-hexanol | B10 | 0.733 |
| 96 | 3-Methyl-1-butanol | B1 | -0.714 |
| 96 | 3-Methyl-1-butanol | B13 | -0.764 |
| 96 | 3-Methyl-1-butanol | B2 | -0.905 |
| 96 | 3-Methyl-1-butanol | B9 | 0.905 |
| 97 | 3-Octanol | B2 | -0.764 |
| 97 | 3-Octanol | F10 | 0.873 |
| 97 | 3-Octanol | F23 | 0.768 |
| 100 | 6-Undecanol | B1 | -0.833 |
| 100 | 6-Undecanol | B13 | -0.764 |
| 100 | 6-Undecanol | B9 | 0.905 |
| 101 | beta-Ethylphenethyl alcohol | B2 | -0.731 |
| 102 | 2-Pentanol | B2 | -0.873 |
| 102 | 2-Pentanol | F10 | 0.764 |
| 102 | 2-Pentanol | F23 | 0.878 |
| 106 | Phenylethyl alcohol | B2 | -0.786 |
| 108 | 2,6-Dimethyl-4-heptanol | B1 | -0.791 |
| 108 | 2,6-Dimethyl-4-heptanol | B9 | 0.791 |
| 108 | 2,6-Dimethyl-4-heptanol | F2 | 0.846 |
| 108 | 2,6-Dimethyl-4-heptanol | F5 | -0.736 |
| 112 | (E)-2-Octen-1-ol | B13 | 0.756 |
| 115 | 1-Octen-3-ol | B13 | 0.756 |
| 116 | Tris(1-methylethyl)silanol | B1 | -0.903 |
| 116 | Tris(1-methylethyl)silanol | B9 | 0.732 |
| 117 | 1-Octanol | F10 | 0.707 |
| 117 | 1-Octanol | F23 | 0.741 |
| 118 | Ethanol | B13 | -0.764 |
| 118 | Ethanol | B14 | -0.905 |
| 118 | Ethanol | B7 | -0.881 |
| 118 | Ethanol | B8 | -0.958 |
| 119 | 2-Methyl-1-propanol | F10 | 0.781 |
| 119 | 2-Methyl-1-propanol | F23 | 0.761 |
| 120 | 2-Ethyl-1-hexanol | B10 | -0.922 |
| 120 | 2-Ethyl-1-hexanol | F2 | 0.731 |
| 120 | 2-Ethyl-1-hexanol | F5 | -0.886 |
| 120 | 2-Ethyl-1-hexanol | F6 | -0.723 |
| 122 | 1-Decanol | B14 | -0.791 |
| 122 | 1-Decanol | B8 | -0.782 |
| 122 | 1-Decanol | F23 | 0.768 |
| 123 | 2,4-Dimethylpentanoic acid | B1 | -0.714 |
| 123 | 2,4-Dimethylpentanoic acid | B13 | -0.764 |
| 123 | 2,4-Dimethylpentanoic acid | B2 | -0.81 |
| 123 | 2,4-Dimethylpentanoic acid | B9 | 0.905 |
| 126 | 2-Ethyl-2-hydroxybutyric acid | B2 | -0.878 |
| 126 | 2-Ethyl-2-hydroxybutyric acid | B9 | 0.805 |
| 127 | 4-Methylpentanoic acid | F23 | 0.898 |
| 131 | Acetic acid | B1 | -0.714 |
| 131 | Acetic acid | B13 | -0.764 |
| 131 | Acetic acid | B2 | -0.905 |
| 131 | Acetic acid | B9 | 0.905 |
| 133 | Heptanoic acid | B10 | -0.81 |
| 133 | Heptanoic acid | F5 | -0.786 |
| 136 | Hexanoic anhydride | B1 | -0.833 |
| 136 | Hexanoic anhydride | B2 | -0.786 |
| 136 | Hexanoic anhydride | B9 | 0.833 |
| 136 | Hexanoic anhydride | F2 | 0.81 |
| 136 | Hexanoic anhydride | F5 | -0.762 |
| 136 | Hexanoic anhydride | F6 | -0.719 |
| 137 | Nonanoic acid | B2 | -0.81 |
| 138 | Undecanoic acid | B8 | -0.753 |
| 139 | Pentanoic acid | B10 | -0.738 |
| 139 | Pentanoic acid | F2 | 0.714 |
| 139 | Pentanoic acid | F5 | -0.81 |
| 140 | Octanoic acid | F5 | -0.714 |
| 142 | 2-Ethylhexanoic acid | B8 | -0.753 |
| 143 | n-Decanoic acid | B2 | -0.81 |
| 143 | n-Decanoic acid | F6 | -0.731 |
| 145 | 2-Nonanone | B14 | -0.761 |
| 145 | 2-Nonanone | B2 | -0.799 |
| 145 | 2-Nonanone | F10 | 0.913 |
| 145 | 2-Nonanone | F14 | -0.71 |
| 145 | 2-Nonanone | F23 | 0.868 |
| 146 | 2-Octanone | B8 | 0.707 |
| 148 | 3-Octanone | F10 | 0.764 |
| 148 | 3-Octanone | F14 | -0.764 |
| 151 | Dicyclohexylmethanone | F10 | 0.833 |
| 156 | 3,4-Dimethylbenzaldehyde | B9 | 0.762 |
| 156 | 3,4-Dimethylbenzaldehyde | F6 | -0.719 |
| 158 | Benzaldehyde | B13 | 1 |
| 158 | Benzaldehyde | B14 | 0.733 |
| 158 | Benzaldehyde | B2 | 0.733 |
| 158 | Benzaldehyde | B7 | 0.764 |
| 158 | Benzaldehyde | B8 | 0.768 |
| 158 | Benzaldehyde | B9 | -0.764 |
| 158 | Benzaldehyde | F14 | 0.764 |
| 158 | Benzaldehyde | F17 | 0.768 |
| 162 | Furfural | B13 | 0.756 |
| 164 | Nonanal | B10 | -0.81 |
| 164 | Nonanal | F2 | 0.786 |
| 164 | Nonanal | F5 | -0.786 |
| 165 | 2,4-Di-tert-butylphenol | B1 | -0.857 |
| 165 | 2,4-Di-tert-butylphenol | B9 | 0.714 |
| 165 | 2,4-Di-tert-butylphenol | F2 | 0.929 |
| 165 | 2,4-Di-tert-butylphenol | F5 | -0.929 |
| 165 | 2,4-Di-tert-butylphenol | F6 | -0.826 |
| 168 | 2-Methoxy-5-methylphenol | B2 | -0.786 |
| 168 | 2-Methoxy-5-methylphenol | F5 | -0.738 |
| 169 | 4-Ethylphenol | B13 | -0.764 |
| 169 | 4-Ethylphenol | B9 | 0.881 |
| 169 | 4-Ethylphenol | F14 | -0.714 |
| 169 | 4-Ethylphenol | F17 | -0.85 |
| 170 | 4-Ethyl-2-methoxyphenol | F2 | 0.738 |
| 170 | 4-Ethyl-2-methoxyphenol | F5 | -0.786 |
| 172 | Phenol | B13 | 0.756 |
| 174 | p-Cresol | B10 | -0.738 |
| 174 | p-Cresol | F5 | -0.81 |

**Table S6**. The correlation coefficient between dominant genera and volatile flavor compounds of ZP35S.

| Number of volatile flavor compounds | Volatile flavor compounds | Number  of genera | Correlation  coefficient | |
| --- | --- | --- | --- | --- |
| 1 | 1,1'-Oxybisethanol diacetate | F2 | 0.764 |  |
| 2 | Hexamethylene diacrylate | B9 | 0.714 |  |
| 3 | 2-Methylpentanoic anhydride | B9 | 0.927 |  |
| 3 | 2-Methylpentanoic anhydride | B7 | -0.708 |  |
| 3 | 2-Methylpentanoic anhydride | B14 | -0.927 |  |
| 3 | 2-Methylpentanoic anhydride | B18 | -0.839 |  |
| 3 | 2-Methylpentanoic anhydride | F24 | 0.724 |  |
| 5 | Methyl 2,4-dimethyl-3-oxopentanoate | F13 | 0.764 |  |
| 5 | Methyl 2,4-dimethyl-3-oxopentanoate | F8 | 0.75 |  |
| 6 | 3-Hydroxy-2,2,4-trimethylpentyl isobutyrate | F13 | 0.81 |  |
| 8 | Ethyl 2-hydroxy-3-methylbutanoate | F2 | 0.736 |  |
| 8 | Ethyl 2-hydroxy-3-methylbutanoate | F17 | -0.812 |  |
| 8 | Ethyl 2-hydroxy-3-methylbutanoate | F14 | -0.875 |  |
| 11 | Ethyl 3-phenylpropionate | B9 | 0.833 |  |
| 11 | Ethyl 3-phenylpropionate | B14 | -0.81 |  |
| 11 | Ethyl 3-phenylpropionate | B18 | -0.791 |  |
| 11 | Ethyl 3-phenylpropionate | F24 | 0.755 |  |
| 21 | Ethyl 2-hydroxy-4-methylvalerate | F2 | 0.786 |  |
| 21 | Ethyl 2-hydroxy-4-methylvalerate | F17 | -0.736 |  |
| 21 | Ethyl 2-hydroxy-4-methylvalerate | F14 | -0.85 |  |
| 22 | Ethyl benzoate | F10 | -0.733 |  |
| 23 | Ethyl phenylacetate | F2 | 0.881 |  |
| 23 | Ethyl phenylacetate | F14 | -0.762 |  |
| 23 | Ethyl phenylacetate | F22 | 0.756 |  |
| 26 | Ethyl hydrogen succinate | F2 | 0.781 |  |
| 26 | Ethyl hydrogen succinate | F5 | -0.927 |  |
| 26 | Ethyl hydrogen succinate | F22 | 0.875 |  |
| 27 | Diethyl succinate | B9 | 0.81 |  |
| 27 | Diethyl succinate | B14 | -0.786 |  |
| 27 | Diethyl succinate | B18 | -0.709 |  |
| 27 | Diethyl succinate | F2 | 0.714 |  |
| 30 | Ethyl butyrate | B9 | 0.738 |  |
| 30 | Ethyl butyrate | B14 | -0.714 |  |
| 30 | Ethyl butyrate | B18 | -0.709 |  |
| 30 | Ethyl butyrate | F2 | 0.81 |  |
| 30 | Ethyl butyrate | F14 | -0.714 |  |
| 34 | Ethyl enanthate | F5 | 0.738 |  |
| 34 | Ethyl enanthate | F13 | 0.881 |  |
| 36 | Propyl caproate | F17 | -0.743 |  |
| 36 | Propyl caproate | F10 | 0.85 |  |
| 40 | Ethyl hexanoate | F5 | 0.738 |  |
| 40 | Ethyl hexanoate | F13 | 0.881 |  |
| 45 | Methyl methoxyacetate | F2 | 0.881 |  |
| 45 | Methyl methoxyacetate | F14 | -0.762 |  |
| 45 | Methyl methoxyacetate | F22 | 0.756 |  |
| 48 | Phenylethyl lactate | F2 | 0.756 |  |
| 48 | Phenylethyl lactate | F14 | -0.708 |  |
| 48 | Phenylethyl lactate | F24 | 0.785 |  |
| 52 | Ethyl lactate | B9 | 0.714 |  |
| 52 | Ethyl lactate | B18 | -0.709 |  |
| 52 | Ethyl lactate | F2 | 0.81 |  |
| 52 | Ethyl lactate | F17 | -0.714 |  |
| 52 | Ethyl lactate | F14 | -0.762 |  |
| 53 | R-(+)-isobutyl lactate | B18 | -0.709 |  |
| 53 | R-(+)-isobutyl lactate | F2 | 0.857 |  |
| 53 | R-(+)-isobutyl lactate | F14 | -0.786 |  |
| 54 | Isoamyl lactate | B9 | 0.738 |  |
| 54 | Isoamyl lactate | B14 | -0.714 |  |
| 54 | Isoamyl lactate | B18 | -0.709 |  |
| 54 | Isoamyl lactate | F2 | 0.81 |  |
| 54 | Isoamyl lactate | F14 | -0.714 |  |
| 62 | Ethyl valerate | B9 | 0.976 |  |
| 62 | Ethyl valerate | B7 | -0.833 |  |
| 62 | Ethyl valerate | B14 | -0.952 |  |
| 62 | Ethyl valerate | B18 | -0.873 |  |
| 63 | Diethyl suberate | B9 | 0.714 |  |
| 64 | Ethyl caprylate | F13 | 0.905 |  |
| 65 | Dihydro-5-pentyl-2(3H)-furanone | B9 | 0.976 |  |
| 65 | Dihydro-5-pentyl-2(3H)-furanone | B7 | -0.81 |  |
| 65 | Dihydro-5-pentyl-2(3H)-furanone | B14 | -0.952 |  |
| 65 | Dihydro-5-pentyl-2(3H)-furanone | B18 | -0.791 |  |
| 66 | Ethyl 2-hydroxy-3-phenylpropanoate | B9 | 0.738 |  |
| 66 | Ethyl 2-hydroxy-3-phenylpropanoate | B14 | -0.714 |  |
| 66 | Ethyl 2-hydroxy-3-phenylpropanoate | B18 | -0.709 |  |
| 66 | Ethyl 2-hydroxy-3-phenylpropanoate | F2 | 0.81 |  |
| 66 | Ethyl 2-hydroxy-3-phenylpropanoate | F14 | -0.714 |  |
| 68 | 2-Phenylethyl acetate | B9 | 0.738 |  |
| 68 | 2-Phenylethyl acetate | B14 | -0.714 |  |
| 68 | 2-Phenylethyl acetate | B18 | -0.709 |  |
| 68 | 2-Phenylethyl acetate | F2 | 0.81 |  |
| 68 | 2-Phenylethyl acetate | F14 | -0.714 |  |
| 72 | Hexyl acetate | B9 | 0.857 |  |
| 72 | Hexyl acetate | B14 | -0.833 |  |
| 72 | Hexyl acetate | B18 | -0.791 |  |
| 72 | Hexyl acetate | F17 | -0.762 |  |
| 72 | Hexyl acetate | F24 | 0.731 |  |
| 75 | Isoamyl acetate | F2 | 0.881 |  |
| 75 | Isoamyl acetate | F14 | -0.762 |  |
| 75 | Isoamyl acetate | F22 | 0.756 |  |
| 77 | Ethyl 4-methylvalerate | B9 | 0.791 |  |
| 77 | Ethyl 4-methylvalerate | B14 | -0.791 |  |
| 77 | Ethyl 4-methylvalerate | F24 | 0.768 |  |
| 86 | 1-Nonanol | B9 | 0.738 |  |
| 86 | 1-Nonanol | B7 | -0.952 |  |
| 86 | 1-Nonanol | B14 | -0.714 |  |
| 86 | 1-Nonanol | B18 | -0.791 |  |
| 86 | 1-Nonanol | F17 | -0.714 |  |
| 86 | 1-Nonanol | F10 | 0.714 |  |
| 88 | 2,5-Dimethyl-2,5-hexanediol | B9 | -0.733 |  |
| 88 | 2,5-Dimethyl-2,5-hexanediol | B7 | 0.733 |  |
| 88 | 2,5-Dimethyl-2,5-hexanediol | B14 | 0.764 |  |
| 88 | 2,5-Dimethyl-2,5-hexanediol | B18 | 0.839 |  |
| 88 | 2,5-Dimethyl-2,5-hexanediol | F2 | -0.764 |  |
| 88 | 2,5-Dimethyl-2,5-hexanediol | F17 | 0.733 |  |
| 88 | 2,5-Dimethyl-2,5-hexanediol | F14 | 0.733 |  |
| 93 | 2-Nonanol | F22 | -0.839 |  |
| 96 | 3-Methyl-1-butanol | F5 | 0.762 |  |
| 96 | 3-Methyl-1-butanol | F13 | 0.833 |  |
| 100 | 6-Undecanol | B9 | 0.952 |  |
| 100 | 6-Undecanol | B7 | -0.786 |  |
| 100 | 6-Undecanol | B14 | -0.929 |  |
| 100 | 6-Undecanol | B18 | -0.791 |  |
| 105 | Benzyl alcohol | B9 | 0.857 |  |
| 105 | Benzyl alcohol | B7 | -0.81 |  |
| 105 | Benzyl alcohol | B14 | -0.786 |  |
| 105 | Benzyl alcohol | B18 | -0.736 |  |
| 106 | Phenylethyl alcohol | F13 | 0.738 |  |
| 107 | Propylene glycol | F22 | 0.866 |  |
| 110 | (E)-2-Nonen-1-ol | F2 | 0.862 |  |
| 110 | (E)-2-Nonen-1-ol | F5 | -0.875 |  |
| 110 | (E)-2-Nonen-1-ol | F14 | -0.761 |  |
| 110 | (E)-2-Nonen-1-ol | F22 | 0.884 |  |
| 111 | Trans-2-U0ecen-1-ol | B7 | -0.764 |  |
| 111 | Trans-2-U0ecen-1-ol | F5 | 0.764 |  |
| 113 | 2-Furanmethanol | F2 | 0.736 |  |
| 113 | 2-Furanmethanol | F5 | -0.748 |  |
| 113 | 2-Furanmethanol | F14 | -0.761 |  |
| 113 | 2-Furanmethanol | F22 | 0.884 |  |
| 116 | Tris(1-methylethyl)silanol | B7 | -0.762 |  |
| 116 | Tris(1-methylethyl)silanol | F5 | 0.714 |  |
| 116 | Tris(1-methylethyl)silanol | F13 | 0.786 |  |
| 118 | Ethanol | F2 | 0.714 |  |
| 118 | Ethanol | F17 | -0.857 |  |
| 118 | Ethanol | F14 | -0.738 |  |
| 120 | 2-Ethyl-1-hexanol | F2 | -0.81 |  |
| 120 | 2-Ethyl-1-hexanol | F14 | 0.738 |  |
| 120 | 2-Ethyl-1-hexanol | F22 | -0.927 |  |
| 121 | 1-Heptanol | F2 | -0.71 |  |
| 121 | 1-Heptanol | F5 | 0.875 |  |
| 121 | 1-Heptanol | F22 | -0.806 |  |
| 131 | Acetic acid | B9 | 0.738 |  |
| 131 | Acetic acid | B14 | -0.714 |  |
| 131 | Acetic acid | B18 | -0.791 |  |
| 131 | Acetic acid | F2 | 0.762 |  |
| 131 | Acetic acid | F17 | -0.786 |  |
| 131 | Acetic acid | F14 | -0.786 |  |
| 132 | Butanoic acid | F13 | 0.81 |  |
| 133 | Heptanoic acid | F22 | -0.708 |  |
| 135 | Hexanoic acid | F22 | -0.708 |  |
| 136 | Hexanoic anhydride | B9 | -0.791 |  |
| 136 | Hexanoic anhydride | B7 | 0.791 |  |
| 136 | Hexanoic anhydride | B14 | 0.846 |  |
| 136 | Hexanoic anhydride | B18 | 0.906 |  |
| 137 | Nonanoic acid | F8 | -0.791 |  |
| 139 | Pentanoic acid | F5 | 0.714 |  |
| 146 | 2-Octanone | B9 | -0.881 |  |
| 146 | 2-Octanone | B14 | 0.857 |  |
| 146 | 2-Octanone | B18 | 0.709 |  |
| 154 | 2,4,5-Trimethylbenzaldehyde | F9 | 1 |  |
| 156 | 3,4-Dimethylbenzaldehyde | F2 | 0.714 |  |
| 159 | Benzeneacetaldehyde | F22 | -0.781 |  |
| 165 | 2,4-Di-tert-butylphenol | B9 | 0.81 |  |
| 165 | 2,4-Di-tert-butylphenol | B14 | -0.714 |  |
| 165 | 2,4-Di-tert-butylphenol | F13 | 0.714 |  |
| 170 | 4-Ethyl-2-methoxyphenol | B9 | 0.881 |  |
| 170 | 4-Ethyl-2-methoxyphenol | B14 | -0.857 |  |
| 170 | 4-Ethyl-2-methoxyphenol | B18 | -0.709 |  |
| 173 | 2-(1,1-dimethylethyl)-4-methylphenol | F5 | -0.738 |  |
| 173 | 2-(1,1-dimethylethyl)-4-methylphenol | F22 | 0.805 |  |
| 174 | p-Cresol | B9 | 0.714 |  |

**Table S7**. The correlation coefficient between dominant genus and volatile flavor compounds of ZP100S.

| Number of volatile flavor compounds | Volatile flavor compounds | Number  of genera | Correlation  coefficient |
| --- | --- | --- | --- |
| 2 | Hexamethylene diacrylate | F17 | -0.731 |
| 2 | Hexamethylene diacrylate | F11 | 0.738 |
| 2 | Hexamethylene diacrylate | F22 | 0.771 |
| 3 | 2-Methylpentanoic anhydride | F20 | 0.85 |
| 3 | 2-Methylpentanoic anhydride | F12 | 0.826 |
| 6 | 3-Hydroxy-2,2,4-trimethylpentyl isobutyrate | B17 | 0.714 |
| 6 | 3-Hydroxy-2,2,4-trimethylpentyl isobutyrate | F17 | -0.743 |
| 6 | 3-Hydroxy-2,2,4-trimethylpentyl isobutyrate | F10 | 0.905 |
| 6 | 3-Hydroxy-2,2,4-trimethylpentyl isobutyrate | F23 | 0.738 |
| 11 | Ethyl 3-phenylpropionate | B5 | 0.857 |
| 11 | Ethyl 3-phenylpropionate | F7 | 0.786 |
| 11 | Ethyl 3-phenylpropionate | F4 | 0.755 |
| 11 | Ethyl 3-phenylpropionate | F23 | 0.762 |
| 13 | Ethyl 3-methylthiopropionate | B13 | -0.85 |
| 13 | Ethyl 3-methylthiopropionate | B17 | 0.903 |
| 13 | Ethyl 3-methylthiopropionate | B12 | 0.83 |
| 13 | Ethyl 3-methylthiopropionate | B5 | 0.927 |
| 13 | Ethyl 3-methylthiopropionate | F16 | 0.805 |
| 14 | Ethyl 3-hydroxyoctanoate | F15 | 0.733 |
| 14 | Ethyl 3-hydroxyoctanoate | F3 | 0.733 |
| 14 | Ethyl 3-hydroxyoctanoate | F2 | -0.733 |
| 20 | Ethyl dl-2-hydroxycaproate | B14 | -0.81 |
| 20 | Ethyl dl-2-hydroxycaproate | F17 | -0.994 |
| 20 | Ethyl dl-2-hydroxycaproate | F10 | 0.881 |
| 20 | Ethyl dl-2-hydroxycaproate | F23 | 0.738 |
| 22 | Ethyl benzoate | F12 | 0.805 |
| 23 | Ethyl phenylacetate | B15 | 0.833 |
| 23 | Ethyl phenylacetate | B17 | 0.762 |
| 23 | Ethyl phenylacetate | B12 | 0.786 |
| 23 | Ethyl phenylacetate | B5 | 0.81 |
| 23 | Ethyl phenylacetate | F7 | 0.881 |
| 23 | Ethyl phenylacetate | F23 | 0.714 |
| 24 | 1-Methoxy-2-propyl acetate | F15 | 0.733 |
| 24 | 1-Methoxy-2-propyl acetate | F3 | 0.733 |
| 24 | 1-Methoxy-2-propyl acetate | F2 | -0.733 |
| 26 | Ethyl hydrogen succinate | B14 | -0.731 |
| 26 | Ethyl hydrogen succinate | F17 | -0.795 |
| 27 | Diethyl succinate | B15 | 0.833 |
| 27 | Diethyl succinate | B17 | 0.786 |
| 27 | Diethyl succinate | B12 | 0.714 |
| 27 | Diethyl succinate | F7 | 0.714 |
| 27 | Diethyl succinate | F10 | 0.762 |
| 28 | beta-Phenylethyl butyrate | B15 | 0.826 |
| 28 | beta-Phenylethyl butyrate | B12 | 0.802 |
| 28 | beta-Phenylethyl butyrate | B5 | 0.862 |
| 28 | beta-Phenylethyl butyrate | F7 | 0.85 |
| 29 | Butyl butyrate | B3 | 0.719 |
| 30 | Ethyl butyrate | B15 | 0.714 |
| 30 | Ethyl butyrate | B17 | 0.762 |
| 30 | Ethyl butyrate | B12 | 0.929 |
| 30 | Ethyl butyrate | B5 | 0.881 |
| 30 | Ethyl butyrate | F7 | 0.881 |
| 30 | Ethyl butyrate | F16 | 0.881 |
| 31 | Isoamyl butyrate | B17 | 0.714 |
| 31 | Isoamyl butyrate | B12 | 0.881 |
| 31 | Isoamyl butyrate | B5 | 0.905 |
| 31 | Isoamyl butyrate | F7 | 0.833 |
| 31 | Isoamyl butyrate | F16 | 0.857 |
| 32 | Butyl butyryllactate | B15 | 0.886 |
| 32 | Butyl butyryllactate | B12 | 0.766 |
| 32 | Butyl butyryllactate | F7 | 0.743 |
| 34 | Ethyl enanthate | B13 | -0.83 |
| 34 | Ethyl enanthate | F4 | 0.719 |
| 36 | Propyl caproate | B3 | 0.714 |
| 36 | Propyl caproate | B15 | 0.786 |
| 36 | Propyl caproate | B12 | 0.714 |
| 36 | Propyl caproate | B5 | 0.738 |
| 36 | Propyl caproate | F7 | 0.762 |
| 36 | Propyl caproate | F12 | 0.786 |
| 38 | Methyl hexoate | B3 | 0.791 |
| 38 | Methyl hexoate | F20 | 0.873 |
| 38 | Methyl hexoate | F11 | 0.846 |
| 38 | Methyl hexoate | F22 | 0.801 |
| 40 | Ethyl hexanoate | B16 | 0.714 |
| 40 | Ethyl hexanoate | B3 | 0.714 |
| 40 | Ethyl hexanoate | B5 | 0.857 |
| 40 | Ethyl hexanoate | F7 | 0.881 |
| 40 | Ethyl hexanoate | F4 | 0.719 |
| 40 | Ethyl hexanoate | F23 | 0.833 |
| 45 | Methyl methoxyacetate | B16 | 0.714 |
| 45 | Methyl methoxyacetate | B3 | 0.905 |
| 45 | Methyl methoxyacetate | F7 | 0.833 |
| 45 | Methyl methoxyacetate | F11 | 0.786 |
| 45 | Methyl methoxyacetate | F23 | 0.738 |
| 46 | Diethyl azelate | B3 | 0.786 |
| 46 | Diethyl azelate | B15 | 0.714 |
| 46 | Diethyl azelate | F17 | -0.707 |
| 46 | Diethyl azelate | F7 | 0.738 |
| 46 | Diethyl azelate | F11 | 0.762 |
| 46 | Diethyl azelate | F22 | 0.771 |
| 47 | Ethyl nonanoate | B13 | -0.839 |
| 47 | Ethyl nonanoate | B17 | 0.846 |
| 47 | Ethyl nonanoate | B12 | 0.791 |
| 47 | Ethyl nonanoate | B5 | 0.764 |
| 48 | Phenylethyl lactate | F10 | 0.786 |
| 48 | Phenylethyl lactate | F23 | 0.833 |
| 49 | Propyl lactate | B15 | 0.762 |
| 49 | Propyl lactate | F17 | -0.802 |
| 49 | Propyl lactate | F4 | 0.731 |
| 52 | Ethyl lactate | F17 | -0.97 |
| 52 | Ethyl lactate | F10 | 0.905 |
| 53 | R-(+)-isobutyl lactate | B14 | -0.83 |
| 53 | R-(+)-isobutyl lactate | F17 | -0.798 |
| 54 | Isoamyl lactate | F17 | -0.85 |
| 54 | Isoamyl lactate | F11 | 0.714 |
| 54 | Isoamyl lactate | F10 | 0.905 |
| 54 | Isoamyl lactate | F23 | 0.714 |
| 57 | Pentyl 2-hydroxypropanoate | F17 | -0.874 |
| 57 | Pentyl 2-hydroxypropanoate | F11 | 0.714 |
| 57 | Pentyl 2-hydroxypropanoate | F10 | 0.81 |
| 57 | Pentyl 2-hydroxypropanoate | F4 | 0.707 |
| 57 | Pentyl 2-hydroxypropanoate | F23 | 0.81 |
| 59 | Diethyl glutarate | B14 | -0.873 |
| 59 | Diethyl glutarate | B7 | -0.87 |
| 62 | Ethyl valerate | B7 | -0.795 |
| 62 | Ethyl valerate | B12 | 0.905 |
| 62 | Ethyl valerate | B5 | 0.81 |
| 62 | Ethyl valerate | F16 | 0.857 |
| 63 | Diethyl suberate | B14 | -0.905 |
| 63 | Diethyl suberate | F17 | -0.934 |
| 63 | Diethyl suberate | F10 | 0.786 |
| 64 | Ethyl caprylate | B15 | 0.81 |
| 64 | Ethyl caprylate | B12 | 0.714 |
| 64 | Ethyl caprylate | B5 | 0.762 |
| 64 | Ethyl caprylate | F7 | 0.857 |
| 64 | Ethyl caprylate | F4 | 0.755 |
| 64 | Ethyl caprylate | F23 | 0.81 |
| 65 | Dihydro-5-pentyl-2(3H)-furanone | B16 | 0.762 |
| 65 | Dihydro-5-pentyl-2(3H)-furanone | B3 | 0.857 |
| 65 | Dihydro-5-pentyl-2(3H)-furanone | F17 | -0.719 |
| 65 | Dihydro-5-pentyl-2(3H)-furanone | F7 | 0.833 |
| 65 | Dihydro-5-pentyl-2(3H)-furanone | F11 | 0.762 |
| 65 | Dihydro-5-pentyl-2(3H)-furanone | F10 | 0.786 |
| 65 | Dihydro-5-pentyl-2(3H)-furanone | F23 | 0.81 |
| 66 | Ethyl 2-hydroxy-3-phenylpropanoate | B3 | 0.762 |
| 66 | Ethyl 2-hydroxy-3-phenylpropanoate | B15 | 0.738 |
| 66 | Ethyl 2-hydroxy-3-phenylpropanoate | F7 | 0.762 |
| 66 | Ethyl 2-hydroxy-3-phenylpropanoate | F10 | 0.714 |
| 66 | Ethyl 2-hydroxy-3-phenylpropanoate | F22 | 0.819 |
| 68 | 2-Phenylethyl acetate | B3 | 0.786 |
| 68 | 2-Phenylethyl acetate | B15 | 0.714 |
| 68 | 2-Phenylethyl acetate | F17 | -0.707 |
| 68 | 2-Phenylethyl acetate | F7 | 0.738 |
| 68 | 2-Phenylethyl acetate | F11 | 0.762 |
| 68 | 2-Phenylethyl acetate | F22 | 0.771 |
| 70 | Ethyl butanoate | B15 | 0.781 |
| 70 | Ethyl butanoate | B12 | 0.83 |
| 70 | Ethyl butanoate | B5 | 0.732 |
| 70 | Ethyl butanoate | F7 | 0.878 |
| 70 | Ethyl butanoate | F16 | 0.708 |
| 71 | Heptyl acetate | B7 | -0.703 |
| 71 | Heptyl acetate | B12 | 0.755 |
| 71 | Heptyl acetate | B5 | 0.778 |
| 71 | Heptyl acetate | F4 | 0.717 |
| 72 | Hexyl acetate | B16 | 0.81 |
| 72 | Hexyl acetate | B3 | 0.905 |
| 72 | Hexyl acetate | F7 | 0.976 |
| 72 | Hexyl acetate | F23 | 0.786 |
| 73 | Pentyl acetate | B16 | 0.707 |
| 73 | Pentyl acetate | B3 | 0.743 |
| 73 | Pentyl acetate | B15 | 0.731 |
| 73 | Pentyl acetate | B12 | 0.874 |
| 73 | Pentyl acetate | F7 | 0.922 |
| 73 | Pentyl acetate | F16 | 0.85 |
| 74 | Ethyl acetate | B9 | 0.738 |
| 74 | Ethyl acetate | F17 | -0.934 |
| 75 | Isoamyl acetate | B13 | -0.854 |
| 75 | Isoamyl acetate | B17 | 0.738 |
| 75 | Isoamyl acetate | B5 | 0.857 |
| 75 | Isoamyl acetate | F4 | 0.826 |
| 75 | Isoamyl acetate | F23 | 0.857 |
| 78 | Isoamyl isovalerate | F17 | -0.753 |
| 78 | Isoamyl isovalerate | F21 | -0.733 |
| 79 | Isopentyl valerate | B11 | 0.732 |
| 79 | Isopentyl valerate | B6 | 0.732 |
| 79 | Isopentyl valerate | B4 | 0.732 |
| 79 | Isopentyl valerate | F12 | 0.781 |
| 80 | Ethyl palmitate | B3 | 0.762 |
| 80 | Ethyl palmitate | B15 | 0.738 |
| 80 | Ethyl palmitate | F11 | 0.786 |
| 83 | 1,7-Octadien-3-ol | F20 | 0.791 |
| 83 | 1,7-Octadien-3-ol | F11 | 0.846 |
| 83 | 1,7-Octadien-3-ol | F22 | 0.801 |
| 85 | 1-Hexanol | B16 | 0.833 |
| 85 | 1-Hexanol | B3 | 0.952 |
| 85 | 1-Hexanol | F7 | 0.857 |
| 85 | 1-Hexanol | F11 | 0.833 |
| 85 | 1-Hexanol | F23 | 0.762 |
| 86 | 1-Nonanol | B9 | 0.976 |
| 86 | 1-Nonanol | B2 | -0.905 |
| 86 | 1-Nonanol | F11 | 0.833 |
| 92 | 2-Hexanol | F2 | -0.846 |
| 93 | 2-Nonanol | B16 | 0.881 |
| 93 | 2-Nonanol | B3 | 0.881 |
| 93 | 2-Nonanol | B12 | 0.714 |
| 93 | 2-Nonanol | B5 | 0.714 |
| 93 | 2-Nonanol | F7 | 1 |
| 93 | 2-Nonanol | F16 | 0.762 |
| 94 | 2-Nonen-1-ol | F11 | 0.733 |
| 96 | 3-Methyl-1-butanol | B14 | -0.762 |
| 96 | 3-Methyl-1-butanol | F17 | -0.934 |
| 97 | 3-Octanol | B16 | 0.799 |
| 97 | 3-Octanol | B3 | 0.812 |
| 97 | 3-Octanol | F7 | 0.824 |
| 97 | 3-Octanol | F10 | 0.748 |
| 100 | 6-Undecanol | F17 | -0.946 |
| 100 | 6-Undecanol | F10 | 0.905 |
| 100 | 6-Undecanol | F4 | 0.743 |
| 100 | 6-Undecanol | F23 | 0.833 |
| 102 | 2-Pentanol | B9 | 0.732 |
| 102 | 2-Pentanol | B2 | -0.805 |
| 102 | 2-Pentanol | F11 | 0.732 |
| 104 | dl-Menthol | B3 | -0.761 |
| 104 | dl-Menthol | F22 | -0.822 |
| 106 | Phenylethyl alcohol | B14 | -0.762 |
| 106 | Phenylethyl alcohol | F17 | -0.97 |
| 106 | Phenylethyl alcohol | F10 | 0.81 |
| 108 | 2,6-Dimethyl-4-heptanol | B3 | 0.731 |
| 108 | 2,6-Dimethyl-4-heptanol | F22 | 0.727 |
| 109 | (E)-2-Hepten-1-ol | F17 | -0.753 |
| 109 | (E)-2-Hepten-1-ol | F21 | -0.764 |
| 113 | 2-Furanmethanol | B3 | 0.786 |
| 115 | 1-Octen-3-ol | B9 | -0.761 |
| 115 | 1-Octen-3-ol | B2 | 0.71 |
| 115 | 1-Octen-3-ol | F11 | -0.71 |
| 117 | 1-Octanol | B16 | 0.874 |
| 117 | 1-Octanol | B3 | 0.886 |
| 117 | 1-Octanol | F7 | 0.898 |
| 117 | 1-Octanol | F10 | 0.731 |
| 117 | 1-Octanol | F22 | 0.776 |
| 118 | Ethanol | B9 | 0.714 |
| 118 | Ethanol | B14 | -0.714 |
| 118 | Ethanol | B2 | -0.762 |
| 118 | Ethanol | F17 | -0.934 |
| 118 | Ethanol | F10 | 0.714 |
| 119 | 2-Methyl-1-propanol | B14 | -0.905 |
| 119 | 2-Methyl-1-propanol | F17 | -0.922 |
| 119 | 2-Methyl-1-propanol | F10 | 0.857 |
| 120 | 2-Ethyl-1-hexanol | F12 | -0.762 |
| 121 | 1-Heptanol | B16 | 0.714 |
| 121 | 1-Heptanol | B3 | 0.881 |
| 121 | 1-Heptanol | F7 | 0.714 |
| 121 | 1-Heptanol | F11 | 0.929 |
| 121 | 1-Heptanol | F10 | 0.714 |
| 121 | 1-Heptanol | F22 | 0.723 |
| 126 | 2-Ethyl-2-hydroxybutyric acid | F17 | -0.778 |
| 126 | 2-Ethyl-2-hydroxybutyric acid | F10 | 0.862 |
| 127 | 4-Methylpentanoic acid | B15 | 0.723 |
| 127 | 4-Methylpentanoic acid | F1 | 0.888 |
| 127 | 4-Methylpentanoic acid | F14 | -0.71 |
| 128 | 5-Methylhexanoic acid | B15 | 0.881 |
| 129 | 6-Methylheptanoic acid | B15 | 0.903 |
| 129 | 6-Methylheptanoic acid | B12 | 0.805 |
| 129 | 6-Methylheptanoic acid | F7 | 0.756 |
| 131 | Acetic acid | B3 | 0.786 |
| 131 | Acetic acid | F7 | 0.738 |
| 132 | Butanoic acid | B3 | 0.952 |
| 132 | Butanoic acid | F20 | 0.762 |
| 132 | Butanoic acid | F7 | 0.738 |
| 132 | Butanoic acid | F11 | 0.881 |
| 132 | Butanoic acid | F22 | 0.819 |
| 133 | Heptanoic acid | B15 | 0.857 |
| 133 | Heptanoic acid | F22 | 0.795 |
| 134 | Heptanoic anhydride | B15 | 0.764 |
| 134 | Heptanoic anhydride | F1 | 0.733 |
| 134 | Heptanoic anhydride | F14 | -0.733 |
| 135 | Hexanoic acid | B16 | 0.833 |
| 135 | Hexanoic acid | B3 | 1 |
| 135 | Hexanoic acid | F20 | 0.738 |
| 135 | Hexanoic acid | F7 | 0.881 |
| 135 | Hexanoic acid | F11 | 0.833 |
| 135 | Hexanoic acid | F22 | 0.819 |
| 136 | Hexanoic anhydride | B16 | 0.714 |
| 136 | Hexanoic anhydride | B3 | 0.929 |
| 136 | Hexanoic anhydride | F20 | 0.786 |
| 136 | Hexanoic anhydride | F7 | 0.833 |
| 136 | Hexanoic anhydride | F11 | 0.714 |
| 137 | Nonanoic acid | B16 | 0.762 |
| 137 | Nonanoic acid | B3 | 0.929 |
| 137 | Nonanoic acid | F7 | 0.881 |
| 137 | Nonanoic acid | F22 | 0.843 |
| 139 | Pentanoic acid | B3 | 0.738 |
| 139 | Pentanoic acid | B15 | 0.762 |
| 139 | Pentanoic acid | B12 | 0.762 |
| 139 | Pentanoic acid | F7 | 0.81 |
| 139 | Pentanoic acid | F22 | 0.735 |
| 140 | Octanoic acid | B16 | 0.857 |
| 140 | Octanoic acid | B3 | 0.976 |
| 140 | Octanoic acid | F7 | 0.929 |
| 140 | Octanoic acid | F11 | 0.786 |
| 140 | Octanoic acid | F22 | 0.747 |
| 140 | Octanoic acid | F23 | 0.738 |
| 141 | 3-Methylbutanoic acid | F3 | 0.762 |
| 143 | n-Decanoic acid | B16 | 0.762 |
| 143 | n-Decanoic acid | B3 | 0.929 |
| 143 | n-Decanoic acid | F7 | 0.881 |
| 143 | n-Decanoic acid | F22 | 0.843 |
| 144 | 2-Heptanone | B14 | -0.762 |
| 144 | 2-Heptanone | B7 | -0.795 |
| 144 | 2-Heptanone | B13 | -0.83 |
| 144 | 2-Heptanone | B17 | 0.762 |
| 145 | 2-Nonanone | B16 | 0.786 |
| 145 | 2-Nonanone | B17 | 0.738 |
| 145 | 2-Nonanone | B12 | 0.714 |
| 145 | 2-Nonanone | B5 | 0.905 |
| 145 | 2-Nonanone | F7 | 0.905 |
| 145 | 2-Nonanone | F16 | 0.762 |
| 145 | 2-Nonanone | F23 | 0.738 |
| 146 | 2-Octanone | B12 | 0.714 |
| 151 | Dicyclohexylmethanone | F21 | 0.731 |
| 152 | 6-Methylhept-5-en-2-one | B13 | 0.783 |
| 152 | 6-Methylhept-5-en-2-one | F17 | 0.768 |
| 152 | 6-Methylhept-5-en-2-one | F10 | -0.764 |
| 156 | 3,4-Dimethylbenzaldehyde | B11 | -0.714 |
| 156 | 3,4-Dimethylbenzaldehyde | B6 | -0.714 |
| 156 | 3,4-Dimethylbenzaldehyde | B4 | -0.714 |
| 156 | 3,4-Dimethylbenzaldehyde | F15 | -0.714 |
| 156 | 3,4-Dimethylbenzaldehyde | F12 | -0.738 |
| 156 | 3,4-Dimethylbenzaldehyde | F14 | 0.762 |
| 158 | Benzaldehyde | B11 | 0.714 |
| 158 | Benzaldehyde | B6 | 0.714 |
| 158 | Benzaldehyde | B4 | 0.714 |
| 158 | Benzaldehyde | F20 | 0.714 |
| 158 | Benzaldehyde | F12 | 0.857 |
| 159 | Benzeneacetaldehyde | B3 | -0.714 |
| 159 | Benzeneacetaldehyde | F7 | -0.738 |
| 159 | Benzeneacetaldehyde | F23 | -0.762 |
| 164 | Nonanal | B11 | 0.857 |
| 164 | Nonanal | B6 | 0.857 |
| 164 | Nonanal | B4 | 0.857 |
| 164 | Nonanal | F15 | 0.738 |
| 164 | Nonanal | F12 | 0.857 |
| 167 | Creosol | F3 | 0.786 |
| 167 | Creosol | F22 | 0.783 |
| 170 | 4-Ethyl-2-methoxyphenol | B16 | 0.786 |
| 170 | 4-Ethyl-2-methoxyphenol | B3 | 0.881 |
| 170 | 4-Ethyl-2-methoxyphenol | F7 | 0.905 |
| 170 | 4-Ethyl-2-methoxyphenol | F11 | 0.714 |
| 170 | 4-Ethyl-2-methoxyphenol | F10 | 0.762 |
| 170 | 4-Ethyl-2-methoxyphenol | F23 | 0.786 |
| 172 | Phenol | F21 | 0.736 |
| 173 | 2-(1,1-dimethylethyl)-4-methylphenol | F3 | 0.833 |
| 173 | 2-(1,1-dimethylethyl)-4-methylphenol | F17 | -0.707 |
| 173 | 2-(1,1-dimethylethyl)-4-methylphenol | F10 | 0.786 |
| 174 | p-Cresol | B9 | 0.714 |
| 174 | p-Cresol | B3 | 0.738 |
| 174 | p-Cresol | F20 | 0.857 |
| 174 | p-Cresol | F11 | 0.857 |
| 177 | Tetradecane | B9 | 0.764 |
| 177 | Tetradecane | B2 | -0.764 |

**Table S8**. The correlation coefficient of the dominant genera in ZP5S.

| Node1 | Node2 | Correlation coefficient |
| --- | --- | --- |
| *Staphylococcus* | *Lactobacillus* | -0.764 |
| *Bacillus* | *Lactobacillus* | -0.857 |
| *Acetobacter* | *Lactobacillus* | -0.81 |
| *Issatchenkia* | *Limosilactobacillus* | 0.786 |
| *Aspergillus* | *Limosilactobacillus* | -0.714 |
| *Kroppenstedtia* | *Thermoactinomyces* | 0.881 |
| *Staphylococcus* | *Thermoactinomyces* | 0.733 |
| *Lacticaseibacillus* | *Thermoactinomyces* | 0.862 |
| *Pichia* | *Thermoactinomyces* | -0.714 |
| *unclassified_o__Saccharomycetales* | *Thermoactinomyces* | -0.79 |
| *Staphylococcus* | *Kroppenstedtia* | 0.764 |
| *Lacticaseibacillus* | *Kroppenstedtia* | 0.766 |
| *Saccharomycopsis* | *Kroppenstedtia* | 0.81 |
| *Bacillus* | *Staphylococcus* | 0.733 |
| *Lacticaseibacillus* | *Staphylococcus* | 0.768 |
| *Thermomyces* | *Staphylococcus* | 0.768 |
| *Saccharomycopsis* | *Staphylococcus* | 0.764 |
| *Pichia* | *Bacillus* | -0.714 |
| *unclassified_o__Saccharomycetales* | *Bacillus* | -0.814 |
| *Aspergillus* | *Acetobacter* | -0.762 |
| *Saccharomycopsis* | *Pichia* | -0.81 |
| *unclassified_o__Saccharomycetales* | *Pichia* | 0.826 |
| *Aspergillus* | *Issatchenkia* | -0.905 |
| *Kazachstania* | *Issatchenkia* | 0.898 |
| *Staphylococcus* | *Thermomyces* | 0.768 |
| *Saccharomycopsis* | *Thermomyces* | 0.814 |

**Table S9**. The correlation coefficient of the dominant genera in ZP35S.

| Node1 | Node2 | Correlation coefficient |
| --- | --- | --- |
| *Kroppenstedtia* | *Lactobacillus* | -0.857 |
| *Thermoactinomyces* | *Lactobacillus* | -0.976 |
| *Virgibacillus* | *Lactobacillus* | -0.873 |
| *Thermoactinomyces* | *Kroppenstedtia* | 0.833 |
| *Virgibacillus* | *Kroppenstedtia* | 0.873 |
| *Virgibacillus* | *Thermoactinomyces* | 0.846 |
| *Saccharomycopsis* | *Aspergillus* | -0.905 |
| *unclassified_f__Teratosphaeriaceae* | *Aspergillus* | 0.756 |
| *unclassified_f__Teratosphaeriaceae* | *Issatchenkia* | -0.708 |
| *Pichia* | *Thermomyces* | -0.786 |
| *Saccharomycopsis* | *Thermomyces* | 0.81 |

**Table S10**. The correlation coefficient of the dominant genera in ZP100S.

| Node1 | Node2 | Correlation coefficient |
| --- | --- | --- |
| *Bacillus* | *Lactobacillus* | -0.881 |
| *Pseudallescheria* | *Lactobacillus* | 0.81 |
| *Intrasporangium* | *Methylobacter* | 1 |
| *Ciceribacter* | *Methylobacter* | 1 |
| *Scedosporium* | *Methylobacter* | 0.762 |
| *Pseudeurotium* | *Methylobacter* | 0.881 |
| *Caproiciproducens* | *unclassified_f__Anaerolineaceae* | 0.833 |
| *Monascus* | *unclassified_f__Anaerolineaceae* | 0.881 |
| *unclassified_f__Teratosphaeriaceae* | *unclassified_f__Anaerolineaceae* | 0.711 |
| *Talaromyces* | *unclassified_f__Anaerolineaceae* | 0.714 |
| *Kroppenstedtia* | *Thermoactinomyces* | 0.771 |
| *Thermomyces* | *Thermoactinomyces* | 0.778 |
| *Pichia* | *Thermoactinomyces* | -0.762 |
| *unclassified_f__Microascaceae* | *Caproiciproducens* | 0.738 |
| *Monascus* | *Caproiciproducens* | 0.881 |
| *Pseudallescheria* | *Caproiciproducens* | 0.833 |
| *unclassified_f__Teratosphaeriaceae* | *Caproiciproducens* | 0.819 |
| *Ciceribacter* | *Intrasporangium* | 1 |
| *Scedosporium* | *Intrasporangium* | 0.762 |
| *Pseudeurotium* | *Intrasporangium* | 0.881 |
| *Rummeliibacillus* | *unclassified_c__Dojkabacteria* | 0.762 |
| *Staphylococcus* | *unclassified_f__Bacteroidetes_vadinHA17* | -0.903 |
| *Staphylococcus* | *Clostridium_sensu_stricto_1* | -0.854 |
| *Pseudallescheria* | *Bacillus* | -0.714 |
| *Rummeliibacillus* | *unclassified_f__Bacteroidetes_vadinHA17* | 0.786 |
| *Clostridium_sensu_stricto_1* | *unclassified_f__Bacteroidetes_vadinHA17* | 0.81 |
| *Talaromyces* | *unclassified_f__Bacteroidetes_vadinHA17* | 0.762 |
| *Scedosporium* | *Ciceribacter* | 0.762 |
| *Pseudeurotium* | *Ciceribacter* | 0.881 |
| *Clostridium_sensu_stricto_1* | *Rummeliibacillus* | 0.762 |
| *Monascus* | *Rummeliibacillus* | 0.714 |
| *Talaromyces* | *Rummeliibacillus* | 0.905 |
| *Monascus* | *Clostridium_sensu_stricto_1* | 0.714 |
| *Pseudeurotium* | *Clostridium_sensu_stricto_2* | 0.714 |
| *Byssochlamys* | *Scedosporium* | 0.857 |
| *Apiotrichum* | *Scedosporium* | 0.762 |
| *Saccharomycopsis* | *Scedosporium* | -0.714 |
| *Pseudeurotium* | *unclassified_f__Microascaceae* | 0.714 |
| *Pseudallescheria* | *unclassified_f__Microascaceae* | 0.786 |
| *unclassified_f__Teratosphaeriaceae* | *unclassified_f__Microascaceae* | 0.759 |
| *Pichia* | *Thermomyces* | -0.862 |
| *Trichosporon* | *unclassified_f__Dipodascaceae* | 0.952 |
| *Talaromyces* | *Monascus* | 0.762 |
| *Saccharomycopsis* | *Apiotrichum* | -0.905 |
| *unclassified_f__Teratosphaeriaceae* | *Pseudallescheria* | 0.711 |
| *unclassified_o__Saccharomycetales* | *Pichia* | 0.738 |
| *unclassified_o__Saccharomycetales* | *Geotrichum* | 0.922 |
